# Annual trends in Mycobacterium tuberculosis detection and rifampicin resistance at a regional referral hospital in Northern Ghana, 2019–2024

**DOI:** 10.1371/journal.pone.0346447

**Published:** 2026-09-18

**Authors:** Solomon Wireko, Joseph Nyarko, Kristine Harley Arthur-Quayson, Osman Abu, Rebecca Appiah, Mina Ofosu, Alexander Kwarteng, John Gameli Deku, Ebenezer Senu

**Affiliations:** 1 Department of Laboratory Technology, Faculty of Health Science, Kumasi Technical University, Kumasi, Ghana; 2 Medicare College of Applied Sciences, Kumasi, Ghana; 3 Upper East Regional Hospital, Bolgatanga, Ghana; 4 Department of Biochemistry and Biotechnology, College of Science, Kwame Nkrumah University of Science and Technology, Kumasi, Ghana; 5 Department of Medical Laboratory Sciences, School of Allied Health Sciences, University of Health and Allied Sciences, Ho, Ghana; 6 Elite Research and Data Science Institute, Kumasi, Ghana; Rutgers Biomedical and Health Sciences, UNITED STATES OF AMERICA

## Abstract

Background

Tuberculosis (TB) remains an important public health problem in Ghana, while rifampicin-resistant TB (RR-TB) poses an additional challenge to TB control. Routine molecular diagnostic data can provide useful information on temporal patterns of TB detection and drug resistance, particularly in regions where population-based surveillance data are limited. However, findings from referral facilities may be influenced by referral patterns and access to diagnostic services and should therefore be interpreted within their health-system context.

We conducted a cross-sectional study using historical GeneXpert MTB/RIF data collected from January 2019 to December 2024 at the Upper East Regional Hospital (UERH), a regional referral facility in Bolgatanga, Ghana. Data were extracted from GeneXpert laboratory and TB programme registers for patients tested for presumptive TB. MTB detection and rifampicin resistance were determined from GeneXpert MTB/RIF results. Temporal differences in categorical outcomes were assessed using Pearson’s chi-square test of independence. Multivariable logistic regression was used to identify factors independently associated with rifampicin resistance.

A total of 6,897 GeneXpert MTB/RIF tests were included. The mean age of the tested population was 44.6 ± 20.4 years, and 56.7% were male. MTB was detected in 1,083 tests (15.7%). MTB detection varied significantly across study years (χ² = 28.46, p < 0.001), with positivity ranging from 9.8% in 2022 to 20.9% in 2021. Among MTB-positive tests, 192/1,083 (17.7%) showed rifampicin resistance. The annual proportion of rifampicin resistance ranged from 9.5% in 2020 to 23.1% in 2023 and varied significantly across years (χ² = 27.13, p < 0.001). Low MTB bacterial load was significantly more frequent among rifampicin-resistant cases. In multivariable analysis, female sex was associated with higher odds of rifampicin resistance after adjustment for age and MTB bacterial-load category (aOR = 1.44, 95% CI: 1.02–2.02; p = 0.037), while low MTB bacterial load was independently associated with rifampicin resistance after adjustment for age and sex (aOR = 9.62, 95% CI: 5.77–16.04; p < 0.001).

MTB detection and rifampicin resistance showed substantial year-to-year variation among patients tested at UERH during 2019–2024. The 17.7% rifampicin-resistance proportion represents a hospital-based estimate among MTB-positive GeneXpert-tested cases and should not be interpreted as the population prevalence of RR-TB in the Upper East Region. The observed pattern nevertheless warrants further investigation to determine the contribution of referral patterns, targeted testing, previous treatment, and other programme factors to the high proportion of rifampicin resistance observed at this referral facility.

## Introduction

Tuberculosis (TB), caused by *Mycobacterium tuberculosis* (MTB), remains one of the leading causes of morbidity and mortality from an infectious disease worldwide. In 2023, an estimated 10.6 million people developed TB globally, with substantial mortality occurring among both people without HIV and people living with HIV [1]. Despite the availability of effective diagnostic and treatment services, TB continues to disproportionately affect low- and middle-income countries, including countries in sub-Saharan Africa, where delays in diagnosis, incomplete access to health services, and gaps in case detection contribute to continued transmission [1–3].

Drug-resistant TB represents an important challenge to TB control. Rifampicin resistance (RR) is an important marker of drug-resistant TB and is frequently used as an indicator for patients requiring further evaluation for multidrug-resistant TB (MDR-TB), which is defined by resistance to at least rifampicin and isoniazid [4]. RR-TB can compromise standard treatment regimens, prolong infectiousness, increase treatment complexity, and contribute to poorer treatment outcomes [4–6]. Rapid identification of rifampicin resistance is therefore an important component of TB surveillance and case management.

The introduction of rapid molecular diagnostic technologies, particularly the Xpert MTB/RIF assay, has substantially improved the ability of TB programmes to detect MTB and rifampicin resistance rapidly. The assay simultaneously detects MTB complex and mutations associated with rifampicin resistance and has been incorporated into TB diagnostic algorithms in many resource-limited settings [7–9]. Nevertheless, access to molecular diagnostics may vary between geographical areas and health facilities because of differences in laboratory infrastructure, referral pathways, equipment functionality, cartridge availability, and programme implementation [10–12]. Consequently, temporal changes in the number of GeneXpert tests performed or the proportion of positive results may reflect both changes in disease occurrence and changes in the health-system pathway through which patients reach diagnostic services.

Ghana remains a country with a substantial TB burden. National surveillance and drug-resistance studies have demonstrated continuing transmission of TB and the presence of drug-resistant disease [13,14]. In Ghana’s first national drug-resistance survey, rifampicin resistance was detected in 2.4% of cultured isolates undergoing drug-susceptibility testing, while MDR-TB was identified in 1.3% of newly diagnosed patients and 25.0% of previously treated patients [15]. These estimates were generated using a national survey design and culture-based drug-susceptibility testing and therefore have a different denominator and methodological basis from routine GeneXpert surveillance conducted at an individual health facility.

The Upper East Region represents an important setting for TB surveillance because it includes predominantly rural populations, geographically dispersed communities, and populations that may experience variable access to diagnostic and treatment services. The Upper East Regional Hospital (UERH), located in Bolgatanga, functions as a regional referral facility and provides diagnostic and treatment services within the Ghana National Tuberculosis Control Programme. Patients evaluated at the hospital may therefore include individuals presenting directly to the facility as well as patients referred from other health facilities. The hospital’s GeneXpert records provide an opportunity to examine patterns in molecular TB detection and rifampicin resistance among patients who reach this referral-level diagnostic service.

However, hospital-based GeneXpert data should not be interpreted as equivalent to population-based surveillance. The number of patients tested at a referral facility may be influenced by referral activity, clinical selection, availability of testing at peripheral facilities, healthcare-seeking behaviour, and programme-specific case-finding activities. Similarly, the proportion of MTB-positive samples that are rifampicin resistant may be affected by selective referral or testing of patients considered to be at increased risk of drug-resistant TB. These considerations are particularly important when interpreting apparently high resistance proportions from a single referral facility.

Published longitudinal data describing routine molecular trends in MTB detection and rifampicin resistance in northern Ghana remain limited. Examining annual GeneXpert data can nevertheless provide useful surveillance information and identify patterns requiring further investigation by the regional and national TB programmes.

Therefore, this study assessed annual trends in MTB detection, bacterial-load distribution, and rifampicin resistance among patients tested using the GeneXpert MTB/RIF assay at the Upper East Regional Hospital from 2019 to 2024. The study was designed to describe patterns within the hospital-based testing population and not to estimate population-level TB incidence or RR-TB prevalence for the Upper East Region.

## Materials and methods

### Study design and setting

A cross-sectional study using historical data collected from January 2019 to December 2024 was conducted at the Upper East Regional Hospital (UERH), a regional referral facility located in Bolgatanga in the Upper East Region of Ghana. Data extraction and analysis were undertaken from Monday, 9 June 2025 to Friday, 29 August 2025.

UERH serves as a regional referral hospital and provides diagnostic and treatment services for TB within the Ghana National Tuberculosis Control Programme. Patients may be referred to the hospital from other health facilities or present directly for evaluation. The hospital therefore represents an important diagnostic site within the regional TB referral network, although its GeneXpert records do not represent all presumptive TB patients or the entire population of the Upper East Region.

### Study population and case definitions

The study population comprised patients evaluated for presumptive TB whose clinical specimens were tested using the GeneXpert MTB/RIF assay at UERH between January 2019 and December 2024.

Presumptive TB was defined as a patient clinically evaluated for possible TB for whom a specimen was submitted for GeneXpert MTB/RIF testing through the hospital’s diagnostic system.

MTB detection was defined as a GeneXpert MTB/RIF result reporting detection of *Mycobacterium tuberculosis* complex.

Rifampicin resistance was defined as detection by GeneXpert MTB/RIF of mutations associated with resistance to rifampicin within the *rpoB* target region. Rifampicin resistance detected by GeneXpert was considered a marker of presumptive RR-TB and did not by itself establish resistance to isoniazid or provide a complete phenotypic drug-susceptibility profile.

Only sputum specimens from patients evaluated for pulmonary TB were included; extrapulmonary TB specimens were not included in the analysis.

GeneXpert MTB/RIF was used as the initial diagnostic test for presumptive pulmonary TB at the Upper East Regional Hospital during the study period. Testing was not restricted or prioritized according to specific patient risk groups. Thus, patients clinically assessed as having presumptive TB were eligible for initial molecular testing using GeneXpert MTB/RIF.

All specimens included in this study were sputum specimens. The GeneXpert MTB/RIF assay was used to simultaneously detect *Mycobacterium tuberculosis* complex and identify molecular markers of rifampicin resistance.

Patients with MTB detected by GeneXpert, including those with rifampicin resistance detected, were referred for culture and drug-susceptibility testing (DST) in accordance with the TB diagnostic and management pathway. GeneXpert-detected rifampicin resistance was therefore considered an indication for further evaluation rather than definitive evidence of multidrug-resistant TB.

### Data collection

Data were extracted from GeneXpert laboratory registers and electronic TB registers. Variables included age, sex, specimen type, MTB detection status, semi-quantitative MTB bacterial-load category, and rifampicin-resistance result.

The semi-quantitative GeneXpert bacterial-load categories were classified as low, moderate, and high according to the result recorded in the laboratory register.

To minimize duplicate observations, repeat GeneXpert tests from the same patient performed within 30 days were excluded from the analysis

### Outcome measures

The primary outcomes were MTB detection and rifampicin resistance among MTB-positive GeneXpert tests.

Secondary outcomes included the distribution of MTB bacterial-load categories and their variation across study years.

### Statistical analysis

Data were analysed using Statistical Package for the Social Sciences (SPSS) version 26.0 (IBM Corp., Armonk, NY, USA) and GraphPad Prism version 9.5. Categorical variables were summarized using frequencies and percentages, while continuous variables were summarized using means and standard deviations.

Differences in categorical outcomes across study years were assessed using Pearson’s chi-square test of independence.

Logistic regression was used to evaluate factors associated with rifampicin resistance among MTB-positive tests. Univariable logistic regression was initially performed, followed by a multivariable logistic regression model including age group, sex, and MTB bacterial-load category. Adjusted odds ratios (aORs) with 95% confidence intervals (CIs) were reported.

In the multivariable model, the association between female sex and rifampicin resistance was adjusted for age group and MTB bacterial-load category, whereas the association between MTB bacterial-load category and rifampicin resistance was adjusted for age group and sex.

Statistical significance was defined as p < 0.05.

### Ethical approval

Ethical approval was obtained from the Institute of Research, Innovation, and Development of Kumasi Technical University (IRID/EC2025/HS0002). Institutional permission was obtained from the Upper East Regional Hospital. Data were anonymized before analysis, and informed consent was waived because the study involved retrospective analysis of routinely collected records.

## Results

### Characteristics of the study population

Between January 2019 and December 2024, 6,897 GeneXpert MTB/RIF tests performed at the Upper East Regional Hospital met the study inclusion criteria. The mean age of the tested population was 44.6 ± 20.4 years. Participants aged 20–39 years constituted the largest age group (33.8%), followed by those aged 40–59 years (30.6%). Males accounted for 3,796 (56.7%) of the tests, while females accounted for 2,902 (43.3%) (Table 1).

**Table 1:** Characteristics of study participants.

Variable	Frequency (n = 6897)	Percentage (%)
**Age (Years) [Mean ±SD]**	**44.64 ± 20.36**	–
Age Group*		
1-19.	684	10.3
20-39	2256	33.8
40-59	2038	30.6
60-95	1693	25.4
Sex*		
Male	3796	56.7
Female	2902	43.3

*: Means variables had missing data

### MTB detection and bacterial-load distribution

MTB was detected in 1,083 of the 6,897 GeneXpert tests, corresponding to an overall MTB positivity of 15.7% among the hospital-based testing population.

Among MTB-positive tests, low bacterial load was the most frequent category, accounting for 46.2% of detected cases, followed by high bacterial load (37.9%) and moderate bacterial load (16.0%).

**Fig 1:**
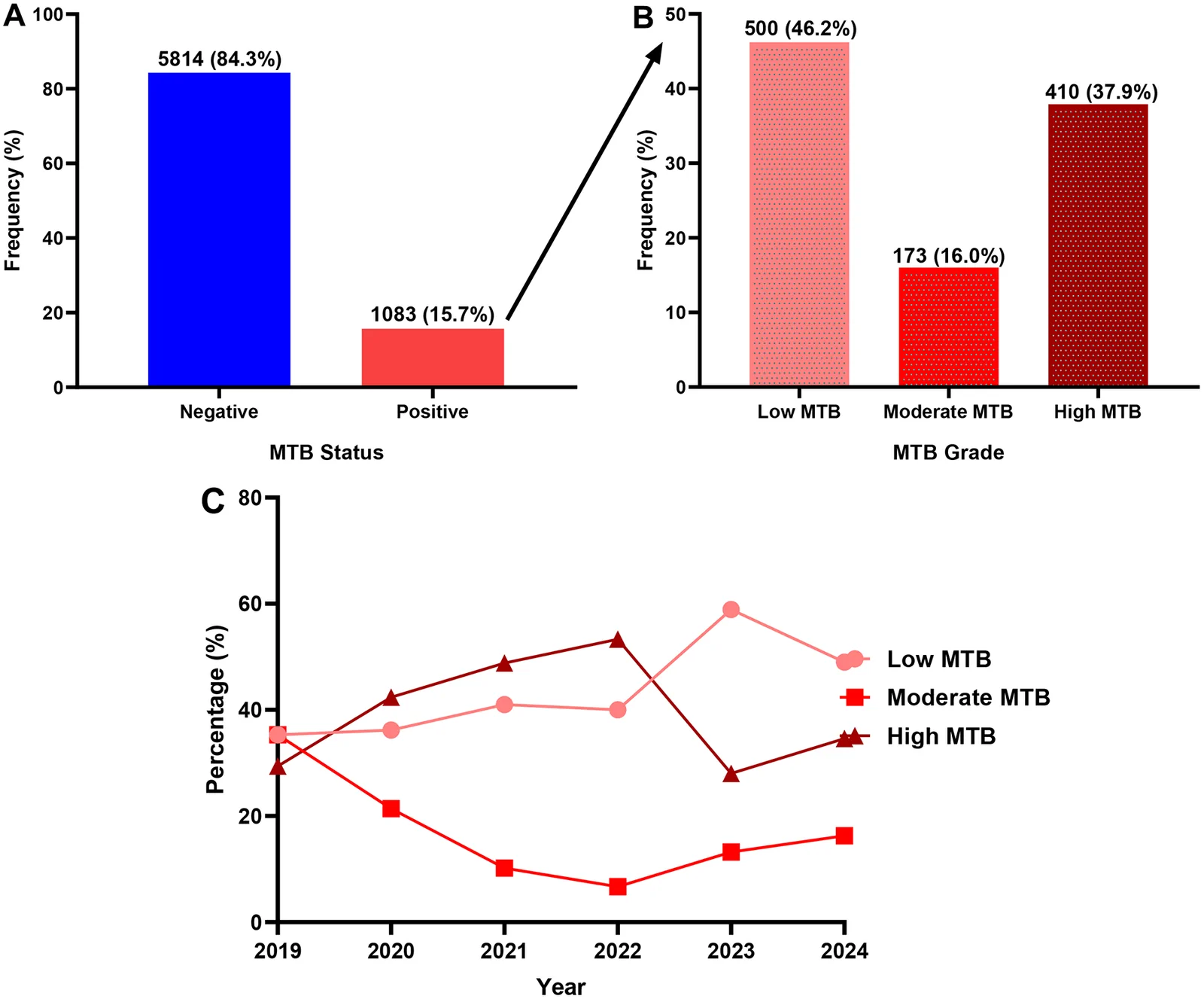
Distribution and temporal trends of *Mycobacterium tuberculosis* detection.

The distribution of MTB bacterial-load categories differed significantly across study years (χ² = 60.11, p < 0.001). High bacterial-load results were particularly frequent between 2020 and 2022, reaching 53.3% of MTB-positive tests in 2022. From 2023, the distribution shifted towards lower bacterial-load results, with low bacterial load accounting for 58.9% of MTB-positive tests in 2023 and 49.0% in 2024 (Fig 1).

### Rifampicin resistance among MTB-positive tests

Rifampicin resistance was detected in 192 of 1,083 MTB-positive tests, representing 17.7% of MTB-confirmed GeneXpert results.

**Fig 2:**
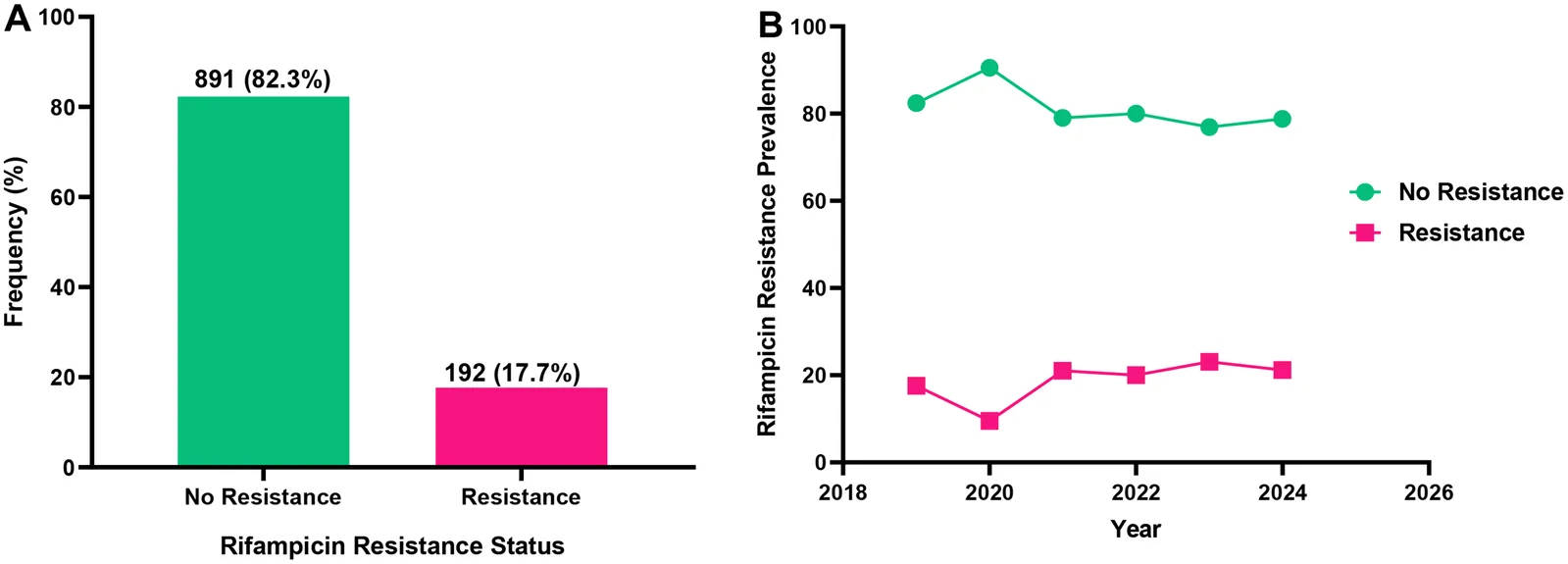
Trend in the proportion of rifampicin resistance among MTB-confirmed cases, 2019–2024.

The annual proportion of rifampicin resistance among MTB-positive tests varied across the study period. The proportion was 17.6% in 2019, decreased to 9.5% in 2020, and subsequently increased to 21.0% in 2021 and 20.0% in 2022. The highest proportion was observed in 2023 (23.1%), followed by 21.2% in 2024. The annual distribution differed significantly across years (χ² = 27.13, p < 0.001) (Fig 2; Table 2).

**Table 2:** Statistical trend of MTB and rifampicin resistance among study participants.

Variable	2019	2020	2021	2022	2023	2024	Test Statistics	*p-value*
**MTB Status**	**n (%)**	**n (%)**	**n (%)**	**n (%)**	**n (%)**	**n (%)**	28.463	**< 0.0001**
Negative	69 (80.2)	2047 (84.7)	776 (79.1)	138 (90.2)	2157 (85.3)	627 (85.8)		
Positive	17 (19.8)	370 (15.3)	205 (20.9)	15 (9.8)	372 (14.7)	104 (14.2)		
**MTB Grade**							60.111	**< 0.0001**
Low	6 (35.3)	134 (36.2)	84 (41.0)	6 (40.0)	219 (58.9)	51 (49.0)		
Moderate	6 (35.3)	79 (21.4)	21 (10.2)	1 (6.7)	49 (13.2)	17 (16.3)		
High	5 (29.4)	157 (42.4)	100 (48.8)	8 (53.3)	104 (28.0)	36 (34.6)		
**Rifampicin Resistance**							27.126	**< 0.0001**
No Resistance	14 (82.4)	335 (90.5)	162 (79.0)	12 (80.0)	286 (76.9)	82 (78.8)		
Resistance	3 (17.6)	35 (9.5)	43 (21.0)	3 (20.0)	86 (23.1)	22 (21.2)		

**Note: p-value computed by Pearson’s chi-square test of independence**

Because the study population consisted of patients tested at a regional referral hospital, these proportions represent rifampicin resistance among MTB-positive GeneXpert-tested patients and should not be interpreted as population-level RR-TB prevalence for the Upper East Region.

### Temporal variation in MTB detection

MTB positivity also varied significantly across study years (χ² = 28.46, p < 0.001). Positivity was highest in 2021 (20.9%) and lowest in 2022 (9.8%). It subsequently increased to 14.7% in 2023 and was 14.2% in 2024 (Table 2).

These findings demonstrate substantial year-to-year variation in the proportion of GeneXpert-tested patients with MTB detected. However, because the study did not include a complete denominator of all presumptive TB patients in the hospital catchment area, these changes cannot be interpreted as direct evidence of corresponding changes in population-level TB incidence.

### Predictors of rifampicin resistance

In univariable logistic regression, female sex, low MTB bacterial load, and moderate MTB bacterial load were associated with rifampicin resistance.

In the multivariable logistic regression model, female sex remained independently associated with rifampicin resistance after adjustment for age group and MTB bacterial-load category (aOR = 1.44; 95% CI: 1.02–2.02; p = 0.037).

Low MTB bacterial load was also independently associated with rifampicin resistance after adjustment for age group and sex (aOR = 9.62; 95% CI: 5.77–16.04; p < 0.001). Moderate bacterial load was not independently associated with rifampicin resistance after adjustment (aOR = 1.91; 95% CI: 0.93–3.95; p = 0.079) (Table 3).

**Table 3:** Predictors of rifampicin resistance among study participants.

Variable	No Resistance (n = 891)	Resistance (n = 192)	*p-value* ^*a*^	cOR (95% CI)	*p-value* ^*b*^	aOR (95% CI)	*p-value* ^*c*^
**Age Group (Years)**	**n (%)**	**n (%)**	0.6700				
1-19.	57 (6.6)	12 (6.5)		1.00	–	1.00	–
20-39	275 (31.9)	61 (33.0)		1.05 (0.53-2.08)	0.8810	1.06 (0.51-2.18)	0.8860
40-59	274 (31.8)	65 (35.1)		1.13 (0.57-2.22)	0.7300	1.14 (0.55-2.34)	0.7310
60-95	256 (29.7)	47 (25.4)		0.87 (0.44-1.75)	0.7000	0.88 (0.42-1.85)	0.7360
**Sex**			**0.0160**				
Male	513 (59.1)	92 (49.5)		1.00	–	1.00	–
Female	355 (40.9)	94 (50.5)		1.48 (1.08-2.03)	**0.0160**	1.44 (1.02-2.02)	**0.0370**
**MTB Grade**			**< 0.0001**				
Low MTB	341 (38.3)	159 (82.8)		10.15 (6.11-16.89)	**< 0.0001**	9.62 (5.77-16.04)	**< 0.0001**
Moderate MTB	158 (17.7)	15 (7.8)		2.07 (1.02-4.20)	**0.0450**	1.91 (0.93-3.95)	0.0790
High MTB	392 (44.0)	18 (9.4)		1.00	–	1.00	–

Superscripts a: p-value computed by Chi-square test, b: p-value computed by Univariable logistic regression model, c: p-value computed by Multivariable logistic regression model.

Abbreviations: cOR: Crude odds ratio, aOR: Adjusted odds ratio, CI: Confidence interval.

The association between low bacterial load and rifampicin resistance should be interpreted cautiously because bacterial load is a laboratory-derived measure and the retrospective dataset did not contain several potentially important clinical and epidemiological variables, including previous TB treatment history and HIV status.

## Discussion

This study examined annual patterns of *Mycobacterium tuberculosis* (MTB) detection, bacterial-load distribution, and rifampicin resistance among patients tested using GeneXpert MTB/RIF at the Upper East Regional Hospital (UERH) between 2019 and 2024. MTB was detected in 15.7% of the 6,897 GeneXpert-tested observations, with substantial variation across years. Rifampicin resistance was detected in 17.7% (192/1,083) of MTB-positive tests, ranging from 9.5% to 23.1% annually. Female sex and low MTB bacterial load were independently associated with rifampicin resistance after adjustment for selected covariates.

The 15.7% MTB positivity should be interpreted as a hospital-based diagnostic proportion rather than a population-level estimate of TB prevalence or incidence. The dataset represents patients who reached a regional referral hospital and underwent GeneXpert testing and therefore does not include all presumptive TB patients in the Upper East Region, persons who did not seek care, or patients tested at other facilities. The observed positivity is nevertheless consistent with findings from hospital-based studies in Ghana and other sub-Saharan African settings, which indicate a substantial burden of MTB among presumptive TB patients presenting for diagnostic evaluation [16–18].

Considerable year-to-year variation in MTB positivity was observed, with positivity increasing to 20.9% in 2021, declining to 9.8% in 2022, and subsequently increasing to 14.7% in 2023 and 14.2% in 2024. These fluctuations should be interpreted as changes in hospital-based detection rather than direct evidence of corresponding changes in population TB incidence. Variations in healthcare utilization, referral patterns, case-finding intensity, diagnostic access, and service availability may contribute to such patterns [19]. The COVID-19 pandemic also disrupted TB diagnostic and treatment services globally and in Africa and may have influenced healthcare-seeking and diagnostic pathways during the study period [1,5,20]. However, because information on annual referral volumes, diagnostic coverage, cartridge availability, equipment downtime, and untested presumptive TB patients was unavailable, the specific causes of the observed temporal variation cannot be established.

A notable change was also observed in MTB bacterial-load distribution, with high bacterial-load results predominating during 2020–2022 and low bacterial-load results becoming more frequent in 2023–2024. This may reflect changes in the characteristics of patients reaching the diagnostic service, referral practices, timing of diagnosis, or case-finding strategies rather than changes in circulating MTB strains. High bacillary loads are generally associated with greater infectiousness, but molecular bacterial-load categories should not be interpreted as direct measures of transmission risk [7,21]. Further investigation would be required to determine the reasons for the observed shift.

The most notable finding was the 17.7% rifampicin-resistance proportion among MTB-positive GeneXpert tests. This figure should not be interpreted as indicating that 17.7% of people with TB in the Upper East Region have RR-TB or as evidence of a regional outbreak. Rather, it represents the proportion of rifampicin-resistant results among MTB-positive tests performed at UERH. The hospital’s role as a regional referral facility may result in enrichment of patients with previous TB treatment, treatment failure, severe disease, or other characteristics associated with referral and drug resistance. Although GeneXpert was used as the initial diagnostic test for presumptive TB and testing was not prioritized for particular drug-resistance risk groups, referral-related differences between patients attending UERH and those managed at peripheral facilities may still influence the observed proportion.

The observed proportion is substantially higher than estimates from Ghana’s national TB drug-resistance survey, which reported rifampicin resistance in 2.4% of cultured isolates undergoing drug-susceptibility testing, with MDR-TB detected in 1.3% of newly diagnosed patients and 25.0% of previously treated patients [15]. Direct numerical comparison is inappropriate because the studies differed in population, sampling strategy, diagnostic methods, and denominators. Nevertheless, the high and persistent proportion observed at UERH, which exceeded 20% in four of the six study years, warrants further investigation. Similar studies from tertiary facilities in Ghana and other sub-Saharan African countries have demonstrated geographical and facility-level variation in drug-resistance patterns [6,16,17,22,23]. Review of treatment history, linkage with TB programme records, culture-based drug-susceptibility testing, and molecular characterization of resistant isolates would help establish the epidemiological significance of this finding.

Low MTB bacterial load was strongly associated with rifampicin resistance in the multivariable analysis (aOR = 9.62; 95% CI: 5.77–16.04). This association may reflect several factors, including previous or partial treatment resulting in reduced bacterial burden while resistant organisms persist [4,6]. Biological differences associated with resistance-conferring *rpoB* mutations and technical characteristics of molecular detection at low MTB concentrations may also contribute [6,7,21,24]. However, the present dataset lacked detailed information on treatment history, clinical status, and culture-based susceptibility results, preventing discrimination between these possible explanations. The finding should therefore be interpreted as an association rather than evidence that low bacterial load causes rifampicin resistance.

Female sex was also independently associated with rifampicin resistance, although the magnitude of association was modest (aOR = 1.44; 95% CI: 1.02–2.02). While TB generally affects males more frequently, sex differences in drug-resistant TB are not consistent across populations [1,20]. The observed association may reflect differences in healthcare-seeking behaviour, referral patterns, previous treatment, household exposure, HIV status, or other unmeasured clinical and social factors rather than a direct biological effect of sex. Because several potentially important confounders were unavailable, this association should not be interpreted causally.

These findings demonstrate the value of routine molecular surveillance while also highlighting the limitations of interpreting laboratory data independently of programme information. GeneXpert provides rapid detection of MTB and rifampicin resistance and has strengthened TB diagnosis and drug-resistance surveillance in resource-limited settings [10–12]. However, changes in positivity or resistance proportions may reflect changes in testing coverage, referral patterns, or diagnostic-system performance rather than changes in disease epidemiology. Integrating laboratory data with information on presumptive TB numbers, referral sources, previous treatment, HIV status, treatment outcomes, diagnostic coverage, and programme indicators would improve interpretation of temporal trends [1,5,22].

This study has several strengths, including the analysis of 6,897 GeneXpert tests over six calendar years and assessment of both MTB detection and rifampicin resistance using a standardized molecular platform. However, the retrospective hospital-based design limits generalizability to the wider Upper East Region. Important explanatory variables, including previous TB treatment, HIV status, referral source, and treatment outcomes, were unavailable. In addition, annual information on testing coverage, cartridge availability, equipment performance, and patients assessed but not tested was unavailable, limiting interpretation of temporal changes. More advanced time-series methods were also not applied; future studies incorporating programme-level data and methods such as segmented regression could provide greater insight into temporal changes and the effects of major health-system disruptions.

Overall, the findings indicate substantial year-to-year variation in MTB detection and a consistently notable proportion of rifampicin resistance among MTB-positive GeneXpert-tested patients at UERH. The 17.7% proportion should be regarded as an important hospital-based surveillance signal requiring verification rather than as a population-level estimate of RR-TB burden or evidence of a regional outbreak. Strengthening integration between laboratory and TB programme surveillance, expanding diagnostic coverage, and confirming suspected drug-resistant cases through appropriate drug-susceptibility testing will be important for improving TB control in the Upper East Region and Ghana more broadly [1,5,22].

## Conclusion

MTB detection and rifampicin resistance showed substantial year-to-year variation among patients tested using GeneXpert MTB/RIF at the Upper East Regional Hospital between 2019 and 2024. MTB positivity ranged from 9.8% to 20.9%, while rifampicin resistance among MTB-positive tests ranged from 9.5% to 23.1%, with an overall proportion of 17.7%.

The findings describe the hospital-based GeneXpert testing population and should not be interpreted as estimates of TB incidence or RR-TB prevalence in the Upper East Region. The relatively high proportion of rifampicin resistance observed at this regional referral facility is nevertheless an important surveillance signal that warrants verification and further investigation. In particular, studies incorporating referral pathways, previous TB treatment, diagnostic coverage, treatment outcomes, and confirmatory drug-susceptibility or molecular data are needed to determine the factors underlying the observed resistance pattern.

Further investigation is also warranted to determine whether the temporal fluctuations in MTB detection primarily reflect changes in referral and diagnostic activity, under-detection, healthcare utilization, or changes in the underlying epidemiology of TB.

### Strengths

This study provides one of the few longitudinal assessments of TB detection and rifampicin resistance trends in northern Ghana using routine programmatic GeneXpert data. The large sample size and six-year study period enabled robust trend analysis and the identification of temporal patterns in TB burden and drug resistance. The use of standardized WHO-recommended molecular diagnostics enhances comparability with national and international surveillance studies.

### Limitations

This study has several limitations that should be considered when interpreting the findings. First, the analysis was based on routinely collected GeneXpert data from a single regional referral hospital. The dataset therefore represents patients who reached the facility and underwent GeneXpert testing and cannot be considered representative of all presumptive TB patients or the general population of the Upper East Region. In particular, the study could not estimate population-level TB incidence or RR-TB prevalence.

Second, the retrospective laboratory dataset did not contain complete information on factors that could explain temporal variation in testing and detection, including referral volumes from peripheral facilities, the total number of presumptive TB patients assessed but not tested by GeneXpert, annual cartridge availability, instrument downtime, and changes in case-finding activities. Consequently, the observed annual fluctuations cannot be attributed to a specific health-system or epidemiological mechanism.

Third, important clinical and epidemiological variables, including previous TB treatment history, HIV status, treatment outcomes, comorbidities, and detailed residential location, were incompletely available. These variables could influence both the likelihood of rifampicin resistance and the probability of referral to a regional facility. Their absence limits interpretation of the observed associations, particularly the associations involving sex and bacterial load.

Fourth, GeneXpert MTB/RIF provides rapid molecular detection of MTB and rifampicin resistance but does not provide a complete phenotypic drug-susceptibility profile. Therefore, the rifampicin-resistance findings should be interpreted as GeneXpert-detected rifampicin resistance and not as confirmation of multidrug resistance unless resistance to isoniazid is also established.

Finally, although the study provides useful temporal surveillance information from a large, routine diagnostic dataset spanning six years, the design does not permit causal explanations of the observed trends. The findings should therefore be regarded as descriptive surveillance signals that can guide further investigation rather than as evidence of changes in population-level TB incidence or transmission.

Repeat GeneXpert tests from the same patient performed within 30 days were excluded to minimize duplicate observations. However, because the study used retrospectively collected routine data, complete identification of all repeat or follow-up tests occurring beyond 30 days could not be assured. Some repeat testing may therefore have remained in the dataset, potentially resulting in minor over-representation of individuals undergoing repeat testing.

## Supporting information

S1 Table Supporting files. (XLSX)
