# Supplementary material for: Annual trends in Mycobacterium tuberculosis detection and rifampicin resistance at a regional referral hospital in Northern Ghana, 2019–2024
Source: PLoS One. 2026 Sep 18;21(9):e0346447. doi: 10.1371/journal.pone.0346447 (PMC13588392; doi:10.1371/journal.pone.0346447)
Supplement: Supplementary file 2 [file pone.0346447.s003.docx]

**Dear Editor,**

**RESPONSE TO REVIEWER COMMENTS**

**Manuscript Title:**
*Temporal Trends in Mycobacterium tuberculosis Detection and Rifampicin Resistance at a Regional Referral Hospital in Northern Ghana, 2019–2024*

We sincerely thank the reviewer for the thorough and constructive comments provided on our manuscript. The suggestions have substantially improved the clarity, methodological rigor, and overall quality of the paper. Below is our detailed point-by-point response to each comment. All revisions have been incorporated into the revised manuscript and highlighted accordingly.

**Reviewer Comment 1: Journal Formatting and Style**

**Comment:**

Please carefully review and align the manuscript with the PLOS ONE Submission Guidelines. Ensure consistency in title capitalization and recheck formatting issues around lines 129 and 167.

**Response:**

Thank you for this important observation. We have carefully revised the manuscript to fully align with the PLOS ONE Submission Guidelines. Specifically:

- Title capitalization has been standardized throughout the manuscript using sentence case formatting.
- Formatting inconsistencies identified around lines 129 and 167 have been corrected and highlighted.
- We additionally reviewed headings, tables, references, spacing, abbreviations, and figure formatting to ensure consistency across the manuscript.

**Reviewer Comment 2: Use of Abbreviations**

**Comment:**

All abbreviations should be written in full at first appearance before using the abbreviated form.

**Response:**

We appreciate this comment. The manuscript has been thoroughly reviewed to ensure that all abbreviations are written in full at first mention before introducing abbreviated forms. For example:

- *Mycobacterium tuberculosis* (MTB)
- Rifampicin-resistant tuberculosis (RR-TB)
- Tuberculosis preventive therapy (TPT)
- This correction has been applied consistently throughout the manuscript.

**Abstract**

**Reviewer Comment 3: Gender Difference in Conclusion**

**Comment:**

The conclusion section does not mention the observed gender difference, despite female sex being associated with rifampicin resistance.

**Response:**

Thank you for highlighting this omission. We have revised the conclusion section of the abstract to include the important finding regarding the association between female sex and rifampicin resistance. The revised conclusion now briefly emphasizes that female sex was independently associated with increased odds of rifampicin resistance (Lines 39-42).

**Methods**

**Reviewer Comment 4: Study Design Terminology**

**Comment:**

Please justify whether the term “retrospective cross-sectional study” is methodologically appropriate. Suggested revision: “A cross-sectional study using historical data collected from 2019–2024.”

**Response:**

We thank the reviewer for this valuable methodological clarification. We agree that the original terminology may create ambiguity regarding the study design. Accordingly, we revised the study design description throughout the manuscript to:

“A cross-sectional study using historical data collected from 2019–2024” (Line 87).

This revised wording more accurately reflects the nature of the analysis while avoiding confusion with retrospective cohort or longitudinal study designs.

**Reviewer Comment 5: Need for Trend or Time-Series Analysis**

**Comment:**

If the study is intended to assess temporal trends, more appropriate inferential trend or time-series analyses may be warranted.

**Response:**

We appreciate this insightful recommendation. Our primary objective was a descriptive assessment of yearly trends in MTB detection and rifampicin resistance using routinely collected diagnostic data rather than formal time-series forecasting or longitudinal modeling. To address the reviewer’s concern:

- We clarified in the Methods section that the trend analyses were descriptive in nature.
- We avoided using terminology suggestive of formal longitudinal or time-series analytical methods.
- Topic has been revised
- We revised figure captions and manuscript wording accordingly.

We also acknowledged in the Discussion (Lines 256-259) and Limitations sections (Lines 284-286) that more advanced time-series analyses could provide additional epidemiological insights in future studies.

**Results Section**

**Reviewer Comment 6: Textual Presentation**

**Comment:**

Suggested revision for line #120:
“Males constituted 56.7% (3,796) of the total tests conducted.”

**Response:**

Thank you for this suggestion. The sentence has been revised as recommended for improved clarity and readability (Line 118).

**Reviewer Comment 7: Table 1 Denominators**

**Comment:**

Please clarify the denominators used for each variable.

**Response:**

We appreciate this important observation regarding data completeness and denominator consistency. Table 1 has been revised to clearly indicate the denominators for each variable separately. Specifically:

- Sex data were available for 6,698 participants.
- Age data were available for 6,671 participants.

The table headings and corresponding percentages have been corrected accordingly to avoid confusion.

**Reviewer Comment 8: Geographic Information**

**Comment:**

If geographic data are available, mapping hotspot areas could support targeted interventions.

**Response:**

Thank you for this excellent suggestion. Unfortunately, detailed geographic variables such as district, village, or residential location were not consistently available in the dataset extracted from the GeneXpert records. We have now explicitly stated this limitation in the revised manuscript and acknowledged that future studies incorporating geospatial analyses could help identify transmission hotspots and inform targeted TB interventions (275-278).

**Reviewer Comment 9: Table 3 Formatting**

**Comment:**

Please check alignment and formatting of superscripts/annotations.

**Response:**

We thank the reviewer for noting this formatting issue. The superscripts and annotations in Table 3 have been corrected and properly aligned in the revised manuscript.

**Reviewer Comment 10: Regression Analysis for MTB Positivity**

**Comment:**

It may also be important to conduct regression analysis for factors associated with MTB positivity.

**Response:**

We appreciate this valuable suggestion. *Due to limitations in available explanatory variables within the dataset, we were unable to perform a robust multivariable analysis for MTB positivity. This limitation has now been acknowledged in the revised manuscript.*

**Figures**

**Reviewer Comment 11: Figure 1**

**Comment:**

Panel “C” alone may be sufficient. Avoid including years 2018, 2025, and 2026. The term “longitudinal line graph” is inappropriate.

**Response:**

We thank the reviewer for these valuable suggestions. While Panel C effectively illustrates the annual changes in MTB grade distribution, we elected to retain Panels A and B because they provide important complementary information on the overall burden of MTB positivity and the cumulative distribution of MTB grades within the study population. Presenting both the aggregate distributions (Panels A and B) alongside the yearly patterns (Panel C) offers readers a more comprehensive understanding of the epidemiological characteristics of MTB in the study setting.

In response to the reviewer's concern, we have removed the years 2018, 2025, and 2026 from the X-axis to improve clarity and have revised the figure title and legend to avoid the use of the term "longitudinal line graph." The figure now describes the annual patterns in MTB grade distribution across the study period.

**Reviewer Comment 12: Figure 2**

**Comment:**

Include all years on the X-axis and reconsider the title wording.

**Response:**

We appreciate the reviewer's suggestion. However, we elected not to include all years on the X-axis because doing so would reduce the clarity and readability of the figure, particularly given the limited plotting space and the number of observations presented. We intended to optimize visual interpretation by emphasizing the overall direction and pattern of rifampicin resistance over the study period as recommended by most researchers and reviewers. Nevertheless, to improve clarity, we have revised the figure title and ensured that the corresponding years are clearly described in the figure legend and Results section.

**Variables and Data Completeness**

**Reviewer Comment 13: Additional Variables**

**Comment:**

Please clarify whether additional variables were available.

**Response:**

Thank you for this important comment. We clarified in the Methods and Limitations sections that several potentially important variables were unavailable in the extracted dataset, including:

- HIV status
- Treatment history
- Type of TB (PTB/EPTB)
- Specimen type
- Urban/rural residence
- District or village of residence

We have now expanded the limitations section to explicitly discuss how the absence of these variables may have affected the depth of analysis and interpretation. (Lines 275-278).

**Discussion**

**Reviewer Comment 14: Summary of Key Findings**

**Comment:**

Please summarize key findings early in the Discussion.

**Response:**

We appreciate this recommendation. The opening section of the Discussion has been revised (Lines 174-200) to include a concise summary of the key findings, including:

- Overall MTB detection burden
- Increasing rifampicin resistance trend
- Association between female sex and rifampicin resistance
- Association with low bacillary load

This provides a clearer context for the subsequent interpretation of findings.

**Reviewer Comment 15: Temporal Trend in MTB Detection**

**Comment:**

Discuss possible explanations for increasing MTB detection up to 2022 followed by decline afterward.

**Response:**

Thank you for this insightful comment. We expanded the Discussion section (Lines 181-200) to explore possible explanations for the observed trend, including:

- Expanded GeneXpert testing capacity and intensified case-finding activities before 2022
- Increased public health attention to TB following disruptions during the COVID-19 pandemic
- Possible post-pandemic reductions in healthcare utilization and TB screening activities
- Changes in referral patterns and diagnostic coverage

We also acknowledged that the observed decline may reflect health-system and epidemiologic dynamics requiring further investigation.

**Reviewer Comment 16: Female Sex and Rifampicin Resistance**

**Comment:**

The observed association between female sex and rifampicin resistance deserves deeper discussion.

**Response:**

We appreciate this important observation. The Discussion section has been expanded to more thoroughly interpret the association between female sex and rifampicin resistance (Line 224-240). We discussed several potential explanations, including:

- Delayed healthcare-seeking behavior among women
- Gender-related barriers to accessing TB services
- Socioeconomic inequalities affecting treatment adherence
- Potential delays in diagnosis and initiation of effective therapy

We also highlighted the public health implications for gender-sensitive TB control strategies and equitable access to diagnostic and treatment services in Ghana.

**References**

**Reviewer Comment 17: Reference Accuracy and Use of Primary Studies**

**Comment:**

Please ensure all statements are appropriately referenced and use primary published studies where possible.

**Response:**

Thank you for this important recommendation. We carefully reviewed and updated the reference list to ensure:

- All statements are appropriately supported by relevant citations.
- Greater use of primary published studies for regional comparisons, including studies from Nigeria, Ethiopia, South Africa, and other Sub-Saharan African countries.
- Consistency in citation formatting according to PLOS ONE guidelines.

All references were cross-checked for accuracy and completeness.

**Closing Statement**

We sincerely thank the reviewer for the detailed and constructive feedback. The comments have significantly strengthened the scientific quality, clarity, and interpretability of the manuscript. We believe the revised manuscript has substantially improved and hope it is now suitable for consideration for publication in PLOS ONE.

Thank you

Solomon Wireko (PHD)

On behalf of all the authors
