# Supplementary material for: Annual trends in Mycobacterium tuberculosis detection and rifampicin resistance at a regional referral hospital in Northern Ghana, 2019–2024
Source: PLoS One. 2026 Sep 18;21(9):e0346447. doi: 10.1371/journal.pone.0346447 (PMC13588392; doi:10.1371/journal.pone.0346447)
Supplement: Supplementary file 3 [file pone.0346447.s002.docx]

***Manuscript Title***

“Temporal Trends in Mycobacterium tuberculosis Detection and Rifampicin Resistance at a Regional Referral Hospital in Northern Ghana, 2019–2024”

***General Comments***

This is a relevant and timely manuscript, particularly given the growing public health concern of rifampicin-resistant tuberculosis in Ghana. The study has the potential to contribute valuable evidence to national TB diagnostic and control interventions. However, the manuscript still requires substantial improvement in several areas, particularly: clearer justification of the study design, more robust statistical and trend analyses, improved presentation of results, and deeper discussion and interpretation of findings.

The discussion section remains relatively shallow and should better contextualize the findings within regional and global TB literature.

***Specific Comments***

1. Journal Formatting and Style

Please carefully review and align the manuscript with the PLOS ONE Submission Guidelines.[PLOS ONE Submission Guidelines.](https://journals.plos.org/plosone/s/submission-guidelines.)

Ensure consistency in title capitalization (use sentence case or title case consistently throughout the manuscript).

Please recheck formatting issues around lines 129 and 167.

2. Use of Abbreviations

All abbreviations should be written in full at first appearance before using the abbreviated form.

For example, Mycobacterium tuberculosis (MTB) should consistently follow this format throughout the manuscript.

Please review the entire manuscript for similar abbreviation inconsistencies.

***Abstract***

The conclusion section does not mention the observed gender difference, despite female sex being associated with rifampicin resistance in the analysis.

This important finding should be briefly highlighted in the conclusion.

***Methods***

Please justify whether the term “retrospective cross-sectional study” is methodologically appropriate and standard for this analysis.

The current terminology may create confusion with retrospective cohort or longitudinal study designs.

I would suggest revising the description to something clearer, such as:

“A cross-sectional study using historical data collected from 2019–2024.”

Furthermore, if the study is intended to assess temporal trends or longitudinal changes, more appropriate inferential trend or time-series analyses may be warranted.

Currently, the analyses presented are primarily consistent with a cross-sectional design.

Please justify whether the term “retrospective cross-sectional study” is methodologically appropriate and standard for this analysis. The current terminology may create confusion with retrospective cohort or longitudinal study designs. I would suggest revising the description to something clearer, such as: “A cross-sectional study using historical data collected from 2019–2024.” Furthermore, if the study is intended to assess temporal trends or longitudinal changes, more appropriate inferential trend or time-series analyses may be warranted. Currently, the analyses presented are primarily consistent with a cross-sectional design.

***Results Section***

Textual Presentation

Line #120:

Current:

“Males constituted 56.7% (n = 3,796) of the study population.”

Suggested revision:

“Males constituted 56.7% (3,796) of the total tests conducted.”

**Table 1**

Since the table contains only two variables, the information could potentially be sufficiently narrated within the text instead of presenting it as a separate table.

If the table is retained:

please clarify the denominators used for each variable.

It appears that age data were available for 6,671 tests and sex data for 6,698 tests, while the table heading states “Frequency (n = 6,897).”

Since percentages are not computed from 6,897 for all variables, the denominator should either be corrected or separately indicated for each variable.

**Geographic Information**

If residential or geographic data are available, it would be valuable to indicate where MTB-positive and rifampicin-resistant cases originated.

Mapping hotspot areas could support targeted interventions such as:

intensified screening,

contact investigation (CI), and

tuberculosis preventive therapy (TPT).

**Table 3**

Please check the alignment and formatting of superscripts/annotations (a, b, c) around line #175.

It may also be important to conduct regression analysis for factors associated with MTB positivity, not only rifampicin resistance.

Figures

**Figure 1**

Panel “C” alone may be sufficient to present the trend clearly.

Please avoid including years 2018, 2025 and 2026 unless updated data are available for those periods.

If such data are available, consider updating the analysis accordingly.

The term “longitudinal line graph” may not be appropriate; simply “line graph” is sufficient.

Otherwise, a true longitudinal or time-series analytical framework should be applied to support the terminology used.

**Figure 2**

Please include all years on the X-axis, as done in Figure 1.

Similarly, avoid including 2018, 2025 and 2026 unless data are available.

Please reconsider the use of terms such as “clinical” and “longitudinal.”

Based on the figure presented, a more appropriate title may be:

“Trend in the proportion of rifampicin resistance among MTB-confirmed TB cases, 2019–2024”. No clinical profile variables appear to be presented in this figure.

Variables and Data Completeness

Please clarify whether additional variables were available in the dataset, such as:

type of TB (PTB/EPTB), treatment history (new vs previously treated), specimen type (sputum, stool, pleural fluid, CSF, synovial fluid, etc.), HIV status, urban/rural residence,

district or village of residence.

If these variables were unavailable, this should be clearly stated as a limitation.

The current limitation section (#224–225) is too brief and does not adequately discuss data limitations.

***Discussion***

After the opening paragraph ( first statement), please briefly summarize the key findings of the study, including: the burden of MTB detection, the prevalence and increasing trend of rifampicin resistance, factors associated with resistance (e.g., female sex and low bacillary load).

Important issues that require further discussion

1. Temporal Trend in MTB Detection

What could explain the increasing MTB detection trend up to 2022 followed by the marked decline afterward?

Possible epidemiologic, programmatic, diagnostic, or health system explanations should be explored.

2. Association Between Female Sex and Rifampicin Resistance

The: the observed association between female sex and rifampicin resistance is important and deserves deeper discussion. If valid, this finding may have implications for:gender equity in TB services, healthcare-seeking behavior, access to diagnosis and treatment, delayed presentation among women in Ghana.

***References***

Please ensure that all statements are appropriately referenced.

Some citations around lines #194–195 may not adequately support the statements presented. Where possible, please use primary published studies for country-specific comparisons, including: Nigeria, Ethiopia, South Africa, and Sub-Saharan Africa.

Please carefully verify reference accuracy and consistency throughout the manuscript.
