# Supplementary material for: Annual trends in Mycobacterium tuberculosis detection and rifampicin resistance at a regional referral hospital in Northern Ghana, 2019–2024
Source: PLoS One. 2026 Sep 18;21(9):e0346447. doi: 10.1371/journal.pone.0346447 (PMC13588392; doi:10.1371/journal.pone.0346447)
Supplement: Supplementary file 4 [file pone.0346447.s004.docx]

**Dear Editor,**

**RESPONSE TO REVIEWER COMMENTS**

**Manuscript Title:
*Annual Trends in Mycobacterium tuberculosis Detection and Rifampicin Resistance at a Regional Referral Hospital in Northern Ghana, 2019–2024***

**Response to Reviewer 1**

We sincerely thank Reviewer 1 for the careful and constructive second-round review of our manuscript entitled **“Annual Trends in Mycobacterium tuberculosis Detection and Rifampicin Resistance at a Regional Referral Hospital in Northern Ghana, 2019–2024.”** We appreciate the reviewer’s recognition of the study's public health importance and the valuable suggestions aimed at improving the epidemiological interpretation, methodological clarity, and generalizability of our findings.

We have carefully considered all comments and have revised the manuscript accordingly. In particular, we have clarified that the study describes **hospital-based GeneXpert testing trends rather than population-level TB incidence**, strengthened the description of the diagnostic and referral context, refined the interpretation of rifampicin resistance, clarified the multivariable logistic regression analysis, improved the presentation of Tables 2 and 3, and revised the conclusion and limitations to ensure that our claims remain proportionate to the study design.

For clarity, each comment is reproduced below, followed by our response and the corresponding changes made to the manuscript.

**Comment 1: Corrections should be carefully addressed in both article and abstracts.**

**Response:**

Thank you for this important observation. We carefully reviewed both the Abstract and the main manuscript to ensure consistency in terminology, study design, interpretation of the findings, and the scope of the conclusions.

In particular, we have revised the Abstract to avoid implying that the observed proportions represent population-level TB incidence or prevalence in the Upper East Region. The revised Abstract now explicitly identifies the study as a retrospective analysis of GeneXpert MTB/RIF records from a regional referral hospital and presents the 17.7% rifampicin-resistance estimate specifically as the proportion observed **among MTB-confirmed samples tested at the hospital** (Lines 45-47).

We have also revised the concluding statement of the Abstract to emphasize temporal variation in hospital-based detection and the need for further investigation rather than making a direct inference about the population-level burden of rifampicin-resistant TB in Northern Ghana (Lines 48- 50).

**Comment 2: Introduction: Authors did not give information about TB/RRTB incidence or burden in the geographical area under study. The apparent 17.7% RR-TB finding should be judged in light of the TB programme structure, under-detection/underreporting, and the terms of reference of other health facilities.**

**Response:**

We agree. The previous version provided national TB estimates but insufficient regional context. We have therefore expanded the Introduction and Discussion to distinguish between:

1. national estimates of TB and MDR/RR-TB;
2. regional/hospital-based surveillance data; and
3. the estimates generated by the present study.

The 17.7% estimate is calculated as 192/1,083 among MTB-positive GeneXpert samples tested at the Upper East Regional Hospital and should therefore not be interpreted as the incidence or prevalence of RR-TB in the Upper East Region or Northern Ghana. The denominator consists of MTB-confirmed samples reaching this particular diagnostic facility and is consequently influenced by referral patterns, case-finding practices, clinical selection of patients for GeneXpert testing, and access to molecular diagnostics.

We have revised the manuscript to make this distinction explicit and to avoid describing the finding as evidence of an outbreak.

The 2021 Ghana Population and Housing Census reports a population of approximately 1.30 million for the Upper East Region, representing approximately 4% of Ghana's population. We have used this information only to provide population context and have explicitly stated that our hospital-based dataset cannot be converted into a regional population prevalence estimate.

We have also expanded the discussion of the National Tuberculosis Control Programme and the role of referral facilities. Because the present dataset does not contain complete information on the number and characteristics of presumptive TB patients managed at every facility in the region, we have avoided claiming that the observed pattern represents the entire regional TB programme.

**Change made:** The Introduction and Discussion now explicitly state that the study provides **hospital-based surveillance evidence** and should not be interpreted as a direct estimate of regional TB incidence or RR-TB prevalence (Lines 98-105 and Lines 112-114)

**Comment 3: The study design should be unified in the article and abstract: “A cross-sectional study using historical data collected from 2019–2024.”**

**Response:**

We agree. The study design has now been made consistent throughout the manuscript and Abstract.

The Methods section currently states:

“A cross-sectional study using historical data collected from 2019–2024…”

We have retained this wording and have ensured that the Abstract uses the same terminology. We have also avoided using “longitudinal study” in places where it could imply prospective follow-up of individuals.

**Change made:** Study design terminology has been standardized throughout the Abstract, Methods, Results, Discussion, and Conclusion (Lines 24-25 and Line 117).

**Comment 4: The case definitions are missing. Define presumptive TB, MTB detection, and rifampicin resistance. Did the study include samples other than sputum or patients with extrapulmonary disease?**

**Response:**
We thank the reviewer for highlighting the need for explicit operational definitions. We have now added definitions of presumptive TB, MTB detection, and rifampicin resistance to the Methods section.

The study included **sputum specimens only**. No other clinical specimen types were included. Consequently, the study population comprised patients evaluated for **presumptive pulmonary TB,** and extrapulmonary TB was not assessed.

Presumptive TB is now defined as a patient clinically evaluated for possible pulmonary TB for whom a sputum specimen was submitted for GeneXpert MTB/RIF testing. MTB detection is defined as a GeneXpert result reporting detection of Mycobacterium tuberculosis complex, while rifampicin resistance is defined as GeneXpert detection of mutations associated with rifampicin resistance within the rpoB target region (Lines 131-139).

We have also clarified that GeneXpert-detected rifampicin resistance represents a molecular marker of rifampicin resistance and does not, by itself, establish resistance to isoniazid or constitute a complete phenotypic drug-susceptibility profile.

**Change made:**
The Methods section has been revised to explicitly state that only sputum specimens were included and that the study therefore evaluated pulmonary TB only (Lines 140-141).

**Comment 5: The importance of this hospital for TB surveillance in Ghana is unclear. How much of the regional population does it represent?**

**Response:**

We appreciate this point and have expanded the description of the study setting.

The Upper East Regional Hospital is a regional referral facility in Bolgatanga and functions as a diagnostic and treatment centre for TB under the Ghana National Tuberculosis Control Programme. The study therefore provides an important source of routine molecular surveillance data for patients referred to or tested at the facility.

However, we agree that the hospital's GeneXpert records cannot be assumed to represent the entire regional population. The Upper East Region had a population of approximately 1.30 million in the 2021 Population and Housing Census, representing approximately 4% of Ghana's population.

We have consequently revised the manuscript to avoid implying that the hospital dataset represents all residents of the Upper East Region.

The revised manuscript now emphasizes that the study provides evidence from a regional referral diagnostic facility, rather than a population-based surveillance system (Lines 121-126).

**Comment 6: Other health facilities in the drainage/catchment area and their role in TB detection and surveillance are insufficiently described.**

**Response:**

We agree that the referral network is important when interpreting the observed temporal variation.

Patients suspected of TB may enter the diagnostic pathway through different levels of the health system, and the number of patients ultimately tested at the regional referral hospital may vary according to referral practices, availability of GeneXpert testing at peripheral facilities, clinical suspicion, and programme-directed case-finding.

The present dataset, however, does not contain sufficiently detailed information on all presumptive TB patients assessed at other health facilities in the region or on the number subsequently referred to the Upper East Regional Hospital. We therefore cannot quantify the proportion of all regional presumptive TB cases captured by this hospital.

We have now explicitly acknowledged this issue and have reframed the interpretation of annual fluctuations as variation in hospital-based diagnostic activity and referral patterns, rather than assuming equivalent changes in the underlying regional incidence of TB (Lines 266- 274).

**Comment 7: Hospital bias is unclear. How representative is the sample of presumptive TB patients attending the hospital?**

**Response:**

We agree that this is an important limitation.

The 6,897 records represent GeneXpert MTB/RIF tests performed at the Upper East Regional Hospital during the study period; they do not represent all individuals who attended the hospital with respiratory symptoms or all persons with presumptive TB in the hospital catchment area.

The denominator therefore represents patients who were selected for and underwent GeneXpert testing, rather than the complete population of presumptive TB patients.

We have revised the Methods and Discussion to make this distinction explicit. We have also clarified that selection for GeneXpert testing may have been influenced by clinical suspicion, referral status, previous TB treatment, risk of drug resistance, or programme-specific testing criteria (266-269).

Accordingly, the observed MTB positivity and RR proportions should be interpreted as testing-population estimates rather than unbiased population estimates.

**Comment 8: How much did GeneXpert cover presumptive TB cases? What was the diagnostic algorithm?**

**Response:**

We thank the reviewer for raising this important issue. We have clarified the diagnostic pathway in the revised Methods (Line 144-147 and Lines 151-155).

At the Upper East Regional Hospital, GeneXpert MTB/RIF was used as the initial diagnostic test for presumptive pulmonary TB, and testing was not prioritized for particular patient groups. All presumptive TB patients who entered the hospital's diagnostic pathway and provided the required sputum specimen were therefore eligible for initial GeneXpert testing.

All specimens included in the present study were sputum specimens. Patients with MTB detected by GeneXpert, including those with rifampicin resistance detected, were subsequently referred for culture and drug-susceptibility testing (DST).

We have also clarified an important distinction regarding GeneXpert coverage. Although GeneXpert was the initial diagnostic test for presumptive TB at UERH, the retrospective dataset does not contain a complete denominator of every person clinically assessed as presumptive TB in the hospital or the wider regional health system. We therefore cannot calculate the proportion of all presumptive TB patients in the Upper East Region who were represented in the dataset.

Because testing was not preferentially restricted to particular high-risk groups, we believe the possibility of deliberate selection of patients based on known risk of drug resistance is reduced. Nevertheless, referral and healthcare-seeking patterns may still influence the composition of patients reaching a regional referral hospital. We have therefore retained this consideration when interpreting the 17.7% rifampicin-resistance proportion.

**Change made:**
The Methods section now explicitly describes GeneXpert MTB/RIF as the initial diagnostic test for presumptive pulmonary TB, states that testing was not prioritized for specific patient groups, confirms that only sputum specimens were included, and describes referral of MTB-positive and rifampicin-resistant cases for culture and DST.

(Lines 142-145 and Line 149-153).

**Comment 9: Could fluctuations be due to GeneXpert cartridge supply, machine functionality, or changes in the referral system?**

**Response:**

We agree that this is an important alternative explanation for the observed temporal variation.

The current dataset does not contain complete machine-maintenance logs, cartridge stock records, downtime records, or annual documentation of referral activation. Therefore, we cannot determine whether the observed changes in the number of tests and MTB detections were caused by changes in cartridge availability, instrument functionality, referral activity, case-finding intensity, or healthcare-seeking behaviour.

We have therefore revised the Discussion to avoid attributing the temporal fluctuations to a specific cause.

The revised interpretation is that the observed fluctuations may reflect a combination of changes in diagnostic access, referral activity, case-finding practices, healthcare utilization, and health-system disruptions, including the COVID-19 period (Lines 266-274).

We now explicitly state that these mechanisms cannot be distinguished using the available retrospective dataset and warrant further investigation.

**Comment 10: “Adjusted multivariate logistic regression but not adjusted OR tests. aOR? Adjusted for what? For females it is adjusted for age and for bacterial load it is adjusted for age and sex.”**

**Response:**

We thank the reviewer for identifying this ambiguity.

We have clarified the statistical analysis and interpretation of the adjusted odds ratios.

The multivariable logistic regression model included **age group, sex, and MTB bacterial-load category**. Accordingly:

- the aOR for **female sex** represents the odds of rifampicin resistance adjusted for age group and MTB bacterial-load category; and
- the aOR for **low MTB grade** represents the odds of rifampicin resistance adjusted for age group and sex.

The previous wording suggesting that the female association was adjusted for sex was incorrect and has been removed.

The revised Results section now states:

“In the multivariable logistic regression model, female sex was associated with higher odds of rifampicin resistance after adjustment for age group and MTB bacterial-load category (aOR = 1.44; 95% CI: 1.02–2.02; p = 0.037). Low MTB bacterial load was independently associated with rifampicin resistance after adjustment for age group and sex (aOR = 9.62; 95% CI: 5.77–16.04; p < 0.001)” (Lines 233-239).

This is also reflected in the revised Table 3 footnote.

**Comment 11: Repeat tests within 30 days for 94 patients were excluded. Is this cross-checking sufficient? Repeat and follow-up GeneXpert tests should be excluded for all enrolled persons.**

**Response:**

We thank the reviewer for this important observation. In the original data-cleaning procedure, repeat GeneXpert tests from the same patient occurring within 30 days were identified and excluded to minimize duplication. A total of 94 such repeat tests were excluded.

We acknowledge that a 30-day criterion may not identify every possible follow-up or repeat test occurring after 30 days. However, because the original retrospective dataset and analysis were constructed using this prespecified data-cleaning criterion, and because complete longitudinal linkage of all patient records beyond this interval was not available, we have retained the original analytical dataset rather than retrospectively modifying the inclusion criteria without the ability to reproduce the complete analysis.

We have clarified this point in the revised Methods and Limitations sections. We also acknowledge that some follow-up tests occurring outside the 30-day interval may not have been identifiable as duplicates, and this should be considered when interpreting the findings.

**Change made:**

The Methods section now states:

“To minimize duplicate observations, repeat GeneXpert tests from the same patient performed within 30 days were excluded from the analysis” (Lines 160-161)

The Limitations section has also been revised to acknowledge the possibility that follow-up tests occurring outside the 30-day interval may not have been identifiable in the retrospective dataset (401 - 405).

**Comment 12: “The test used is chi-square for independence” because there are different types of chi-square tests.**

**Response:**

We agree and have corrected the terminology.

The analysis compared categorical variables across independent annual groups; therefore, the appropriate terminology is Pearson's chi-square test of independence.

The revised Methods and table footnotes now specify:

“Temporal differences in categorical variables across study years were assessed using Pearson's chi-square test of independence” (Lines 172-173)

We have also replaced the generic phrase “chi-square test” with “Pearson's chi-square test of independence” where appropriate.

**Comment 13: Tables 2 and 3 do not express themselves well. Should readers assume that bare numbers are absolute numbers and those in brackets are percentages?**

**Response:**

We agree that the tables should be self-explanatory.

Tables 2 and 3 have been revised so that the presentation explicitly indicates that values are reported as **n (%)**, with the number of observations followed by the percentage in parentheses.

For example:

370 (15.3)

is explicitly identified as **370 observations (15.3%)**.

We have also revised the table headings and footnotes to ensure that readers do not need to infer the meaning of the numbers.

For Table 3, the headings for crude and adjusted odds ratios and their corresponding p-values have also been clarified.

**Comment 14: Conclusion: The main conclusion should concern fluctuation in referred presumptive cases rather than positivity. The fluctuation may reflect referral-system activation, under-detection, or underreporting rather than true changes in positivity.**

**Response:**

We agree with the reviewer and have substantially revised the interpretation of the temporal findings.

The original manuscript may have overemphasized temporal changes in MTB detection without sufficiently distinguishing changes in the number of patients tested from changes in the underlying population burden of TB.

Our data demonstrate significant variation in GeneXpert-tested presumptive TB cases and MTB positivity among those tested, but they cannot determine whether the underlying regional incidence of TB increased or decreased.

We have therefore revised the Discussion and Conclusion to emphasize that fluctuations in the observed hospital-based detection pattern may reflect changes in referral activity, diagnostic access, case-finding, healthcare utilization, and under-detection/underreporting.

We now explicitly state that these data cannot distinguish changes in disease occurrence from changes in the health-system pathway through which presumptive TB patients reached the hospital.

**Comment 15: “Significant temporal variation” should have the direction expressed.**

**Response:**

We agree.

The revised Results section now describes the direction and magnitude of the observed annual variation rather than simply reporting “significant temporal variation.”

For MTB detection, positivity was highest in 2021 (20.9%) and lowest in 2022 (9.8%), followed by an increase to 14.7% in 2023 and 14.2% in 2024.

For rifampicin resistance among MTB-confirmed cases, the proportion ranged from 9.5% in 2020 to 23.1% in 2023, with higher proportions observed from 2021 onward compared with 2020.

We have therefore replaced nonspecific wording such as “significant temporal variation” with descriptions of the actual direction and annual pattern.

**Comment 16: Conclusions should be confined to hospital cases rather than the geographical area because of generalizability issues.**

**Response:**

We fully agree.

The conclusion has been revised so that findings are attributed to patients tested at the Upper East Regional Hospital, rather than to the entire Upper East Region or Northern Ghana (Lines 355-356).

We now explicitly acknowledge that the hospital's referral population may differ from the general population and that the study should not be interpreted as a population-based estimate of TB or RR-TB burden

**Comment 17: The 17.7% RR rate is high and needs thorough discussion before concluding it. It requires urgent action to determine whether the finding is real and why it is high.**

**Response:**

We agree with the reviewer that this finding requires cautious interpretation and further investigation.

The observed 17.7% represents 192 of 1,083 MTB-positive GeneXpert samples and therefore reflects rifampicin resistance among MTB-confirmed samples tested at this regional referral hospital. It does not represent the prevalence or incidence of RR-TB in the regional population.

We have revised the Discussion to place this finding in the context of selection into the GeneXpert-tested population and the possibility that regional referral facilities receive a disproportionate number of patients with complicated disease, previous treatment, suspected drug resistance, or treatment failure (Lines 284-292)

The manuscript also now explicitly acknowledges that GeneXpert MTB/RIF detects rifampicin resistance but does not establish resistance to isoniazid or provide a complete phenotypic drug-susceptibility profile

We therefore no longer characterize the 17.7% estimate as evidence of an RR-TB outbreak.

Nevertheless, we agree that the magnitude of the observed hospital-based estimate is important and warrants verification through additional surveillance, review of patient treatment histories, culture-based drug susceptibility testing where available, and/or molecular characterization.

The revised Discussion therefore recommends investigation to determine whether the observed high proportion reflects a true concentration of RR-TB among patients reaching the referral facility, referral/selection effects, or other programme-related factors.

**Comment 18: Recommendations need not be added to the Abstract, but if retained they should be brief.**

**Response:**

We agree.

The Abstract has been revised to minimize recommendations and focus primarily on the study findings.

Where a forward-looking statement is retained, it has been shortened to emphasize the need for investigation of the observed fluctuations in MTB detection and the unexpectedly high hospital-based proportion of rifampicin resistance.

The revised wording is:

“Further investigation is warranted to determine the causes of fluctuations in MTB detection and to verify the magnitude and determinants of rifampicin resistance among patients tested at the regional referral hospital.”

**Comment 19: The stated limitations are not major limitations, particularly because GeneXpert is highly sensitive and the study aimed to assess temporal trends rather than predictors of detection.**

**Response:**

We appreciate this clarification and agree that some of the limitations in the previous version were framed too broadly.

We have revised the Limitations section to distinguish between limitations that directly affect the temporal surveillance objective and limitations that primarily affect interpretation of individual-level predictors or population generalizability.

The use of GeneXpert MTB/RIF is a strength for detecting MTB and rifampicin resistance in routine diagnostic specimens because it provides standardized molecular testing and allows simultaneous assessment of MTB detection and rifampicin resistance.

The main limitations relevant to this study are therefore:

1. the retrospective, single-facility nature of the dataset;
2. incomplete information on referral pathways, diagnostic coverage, cartridge availability, machine downtime, and other health-system factors that could explain temporal fluctuations;
3. absence of complete clinical variables such as previous TB treatment and HIV status;
4. inability to establish population-level TB incidence or RR-TB prevalence from hospital-based GeneXpert records; and
5. inability of GeneXpert MTB/RIF alone to provide a complete drug-susceptibility profile.

We have removed or reduced limitations that do not materially affect the primary objective of describing temporal patterns in GeneXpert-based MTB detection and rifampicin resistance.

**Additional revisions to the manuscript**

In response to the reviewer's comments, we have also undertaken a broader consistency review of the manuscript. Specifically:

- The Abstract and Methods now use consistent study-design terminology.
- Operational definitions for presumptive TB, MTB detection, and rifampicin resistance have been added.
- The hospital-based nature of the study has been emphasized.
- The distinction between hospital-based RR detection and population-level RR-TB prevalence has been clarified.
- The direction of annual changes in MTB positivity and rifampicin resistance has been explicitly described.
- The terminology “Pearson's chi-square test of independence” has been adopted.
- Tables 2 and 3 have been reformatted to clearly indicate **n (%)**.
- The interpretation of the adjusted odds ratios has been corrected.
- The conclusion has been restricted to the population represented by the dataset.
- The interpretation of the 17.7% rifampicin-resistance finding has been substantially moderated.
- The limitations have been revised to focus on limitations that are directly relevant to the study objectives.
- The need for further investigation of the observed rifampicin-resistance pattern has been emphasized without describing the finding as proof of a regional outbreak.

We thank Reviewer 1 again for these constructive comments. We believe that the revisions have substantially improved the methodological clarity, epidemiological interpretation, and appropriate scope of inference of the manuscript.

Thank you,

Solomon Wireko (PhD)

On behalf of all the authors
