# Supplementary material for: Annual trends in Mycobacterium tuberculosis detection and rifampicin resistance at a regional referral hospital in Northern Ghana, 2019–2024
Source: PLoS One. 2026 Sep 18;21(9):e0346447. doi: 10.1371/journal.pone.0346447 (PMC13588392; doi:10.1371/journal.pone.0346447)
Supplement: Peer review history

# Peer review: Decision Letter 0

**DOI:** 10.1371/journal.pone.0346447.r001

**Authors:** Zewdu Dememew

25 May 2026

Dear Dr. Wireko,

Thank you for submitting your manuscript to PLOS ONE. After careful consideration, we feel that it has merit but does not fully meet PLOS ONE’s publication criteria as it currently stands. Therefore, we invite you to submit a revised version of the manuscript that addresses the points raised during the review process.

Please submit your revised manuscript by Jul 09 2026 11:59PM. If you will need more time than this to complete your revisions, please reply to this message or contact the journal office at plosone@plos.org. When you're ready to submit your revision, log on to https://www.editorialmanager.com/pone/ and select the 'Submissions Needing Revision' folder to locate your manuscript file.

- A letter that responds to each point raised by the academic editor and reviewer(s). You should upload this letter as a separate file labeled 'Response to Reviewers'.
- A marked-up copy of your manuscript that highlights changes made to the original version. You should upload this as a separate file labeled 'Revised Manuscript with Track Changes'.
- An unmarked version of your revised paper without tracked changes. You should upload this as a separate file labeled 'Manuscript'.

If you would like to make changes to your financial disclosure, please include your updated statement in your cover letter. Guidelines for resubmitting your figure files are available below the reviewer comments at the end of this letter.

If applicable, we recommend that you deposit your laboratory protocols in protocols.io to enhance the reproducibility of your results. Protocols.io assigns your protocol its own identifier (DOI) so that it can be cited independently in the future. For instructions see: https://journals.plos.org/plosone/s/submission-guidelines#loc-laboratory-protocols. Additionally, PLOS ONE offers an option for publishing peer-reviewed Lab Protocol articles, which describe protocols hosted on protocols.io. Read more information on sharing protocols at https://plos.org/protocols?utm_medium=editorial-email&utm_source=authorletters&utm_campaign=protocols.

As the corresponding author, your ORCID iD is verified in the submission system and will appear in the published article. PLOS supports the use of ORCID, and we encourage all coauthors to register for an ORCID iD and use it as well. Please encourage your coauthors to verify their ORCID iD within the submission system before final acceptance, as unverified ORCID iDs will not appear in the published article. *Only*  the individual author can complete the verification step; PLOS staff *cannot*  verify ORCID iDs on behalf of authors.

We look forward to receiving your revised manuscript.

Kind regards,

Zewdu Gashu Dememew, M.D, PhD

Academic Editor

PLOS One

Journal Requirements:

When submitting your revision, we need you to address these additional requirements.

1. Please ensure that your manuscript meets PLOS ONE's style requirements, including those for file naming. The PLOS ONE style templates can be found at

https://journals.plos.org/plosone/s/file?id=wjVg/PLOSOne_formatting_sample_main_body.pdf and https://journals.plos.org/plosone/s/file?id=ba62/PLOSOne_formatting_sample_title_authors_affiliations.pdf

2. We note that your Data Availability Statement is currently as follows: All relevant data are within the manuscript and its Supporting Information files.

Please confirm at this time whether or not your submission contains all raw data required to replicate the results of your study. Authors must share the “minimal data set” for their submission. PLOS defines the minimal data set to consist of the data required to replicate all study findings reported in the article, as well as related metadata and methods (https://journals.plos.org/plosone/s/data-availability#loc-minimal-data-set-definition).

For example, authors should submit the following data:

- The values behind the means, standard deviations and other measures reported;

- The values used to build graphs;

- The points extracted from images for analysis.

Authors do not need to submit their entire data set if only a portion of the data was used in the reported study.

If your submission does not contain these data, please either upload them as Supporting Information files or deposit them to a stable, public repository and provide us with the relevant URLs, DOIs, or accession numbers. For a list of recommended repositories, please see https://journals.plos.org/plosone/s/recommended-repositories.

If there are ethical or legal restrictions on sharing a de-identified data set, please explain them in detail (e.g., data contain potentially sensitive information, data are owned by a third-party organization, etc.) and who has imposed them (e.g., an ethics committee). Please also provide contact information for a data access committee, ethics committee, or other institutional body to which data requests may be sent. If data are owned by a third party, please indicate how others may request data access.

3. If the reviewer comments include a recommendation to cite specific previously published works, please review and evaluate these publications to determine whether they are relevant and should be cited. There is no requirement to cite these works unless the editor has indicated otherwise.

[Note: HTML markup is below. Please do not edit.]

Reviewers' comments:

Reviewer's Responses to Questions

**Comments to the Author**

1. Is the manuscript technically sound, and do the data support the conclusions?

Reviewer #1: Yes

Reviewer #2: Yes

**********

2. Has the statistical analysis been performed appropriately and rigorously?

Reviewer #1: Yes

Reviewer #2: Yes

**********

3. Have the authors made all data underlying the findings in their manuscript fully available?

The PLOS Data policy

Reviewer #1: Yes

Reviewer #2: Yes

**********

4. Is the manuscript presented in an intelligible fashion and written in standard English?

Reviewer #1: Yes

Reviewer #2: Yes

**********

Reviewer #1: THE STUDY MISSED TO EXPRESS THE DIAGNOSTIC ALGORITHM, IT THE TEST IS FOR EVERY PULMONARY PRESUMPTIVE TB DISEASE OR USED WITH EVERY BACTERIOLOGICAL CONFIRMATION, OR WHAT WAS THE TOTAL NUMBER OF ALL DIAGNOSED PATIENTS AND WHAT WAS THE PROPORTION OF THE TESTED PATIENTS FOR GX/RIFAMPICIN RESISTANCE OR HOW MUCH THE SAMPLE REPRESENT OUT OF THOSE TESTED OR THE REPRESENTATION OVER THE STUDIED PERIOD, IN OHTER WORDS, THE AUTHORS DID NOT REFLECT HOW MUCH THE SAMPLE REPRESENT THE POPULATION UNDER STUDY ADN HENCE CREATING A GENERALIZABILITY ISSUE.

UNLIKE COHORT STUDIES, USING NEW OR OLD DATA IN CROSS-SECTIONAL STUDIES DOES NOT LABEL THEM AS RETRO-OR PRO-SPECTIVE STUDIES. SO THIS IS A CROSS-SECTIONAL STUDY UTILIZING A SETTING DATABASE RELATED TO THE PERIOD 2019-2024.

THE FLUCTUATION OF NUBER OF TESTED OVER THE YEARS FROM A FEW TO HUNDREDS AND RETURNS TO A FEW, (FEW TB PATIENTS IN YEARS 2019 & 2022) BRINGS A QUESTION ABOUT THE AVAILABILITY OF THE TEST OR DOCUMENTATION OR THE UTILIZATION OF THE HOSPITAL AS A TB MANAGEMENT CENTRE.

WHILE CONCLUSIONS SHOULD REFLECT FINDINGS IN LIGHT OF THE STUDY TITILE AND AIM, AND THE TITLE OF THE STUDY REFERS TO THE TEMPORAL TRENDS, THIS WAS NOT MENTION ABOUT IN CONCLUSIONS AND THE CONCLUSIONS MAINLY CONTAINED RECOMMENDATIONS WHICH ARE OUT THE AIM OF THE STUDY.

AUTHORS MENTIONED A SIGNIFICANT AND IMPORTANT PUBLIC HEALTH TOPICS THAT NEED TO SPEAK ABOUT AND TO DISSEMINATE KNOWLEDGE ABOUT IT AND GAVE MUCH EFFORT ON ANALYSIS AND WRITING. I PREFER TO AVOID ANALYTIC PRESENTATION AND STICK TO OBSERVED DETECTION AND SAMPLES LOAD OVER TIME AS A SIMPLE DESCRIPTION FOR THE WORKLOAD WITH IN TURN REFLECTS A SERIOUS BURDEN TO DISEASE ON THE DRAINAGE AREA FOR THE SETTING UNDER STUDY.

Reviewer #2: The conclusions were drawn from the data presented. The increasing resistance observed over recent years, and the female sex and low bacterial load that were independently associated with rifampicin resistance are a reflection of the data and results from the research. The statistical analysis been performed appropriately and rigourously with rerefence to the Chi-square test for the temporal trends in resistance and the logistic regression regarding the female sex and low bacterial load predictors. The authors indicate that the data is fully available.

**********

what does this mean?). If published, this will include your full peer review and any attached files.

If you choose “no”, your identity will remain anonymous but your review may still be made public.

**Do you want your identity to be public for this peer review?**  For information about this choice, including consent withdrawal, please see our Privacy Policy

Reviewer #1: **Yes:**  LAYTH AL-SALIHI

Reviewer #2: No

**********

[NOTE: If reviewer comments were submitted as an attachment file, they will be attached to this email and accessible via the submission site. Please log into your account, locate the manuscript record, and check for the action link "View Attachments". If this link does not appear, there are no attachment files.]

To ensure your figures meet our technical requirements, please review our figure guidelines: https://journals.plos.org/plosone/s/figures

You may also use PLOS’s free figure tool, NAAS, to help you prepare publication quality figures: https://journals.plos.org/plosone/s/figures#loc-tools-for-figure-preparation.

NAAS will assess whether your figures meet our technical requirements by comparing each figure against our figure specifications.

Attachment Submitted filename: Ghana REF RES_Aced Editor_feedback_May 2026.docx

# Author comment: Author response to Decision Letter 1

**DOI:** 10.1371/journal.pone.0346447.r002

30 Jun 2026

Dear Editor,

RESPONSE TO REVIEWER COMMENTS

Manuscript Title:

Temporal Trends in Mycobacterium tuberculosis Detection and Rifampicin Resistance at a Regional Referral Hospital in Northern Ghana, 2019–2024

We sincerely thank the reviewer for the thorough and constructive comments provided on our manuscript. The suggestions have substantially improved the clarity, methodological rigor, and overall quality of the paper. Below is our detailed point-by-point response to each comment. All revisions have been incorporated into the revised manuscript and highlighted accordingly.

Reviewer Comment 1: Journal Formatting and Style

Comment:

Please carefully review and align the manuscript with the PLOS ONE Submission Guidelines. Ensure consistency in title capitalization and recheck formatting issues around lines 129 and 167.

Response:

Thank you for this important observation. We have carefully revised the manuscript to fully align with the PLOS ONE Submission Guidelines. Specifically:

• Title capitalization has been standardized throughout the manuscript using sentence case formatting.

• Formatting inconsistencies identified around lines 129 and 167 have been corrected and highlighted.

• We additionally reviewed headings, tables, references, spacing, abbreviations, and figure formatting to ensure consistency across the manuscript.

Reviewer Comment 2: Use of Abbreviations

Comment:

All abbreviations should be written in full at first appearance before using the abbreviated form.

Response:

We appreciate this comment. The manuscript has been thoroughly reviewed to ensure that all abbreviations are written in full at first mention before introducing abbreviated forms. For example:

• Mycobacterium tuberculosis (MTB)

• Rifampicin-resistant tuberculosis (RR-TB)

• Tuberculosis preventive therapy (TPT)

• This correction has been applied consistently throughout the manuscript.

Abstract

Reviewer Comment 3: Gender Difference in Conclusion

Comment:

The conclusion section does not mention the observed gender difference, despite female sex being associated with rifampicin resistance.

Response:

Thank you for highlighting this omission. We have revised the conclusion section of the abstract to include the important finding regarding the association between female sex and rifampicin resistance. The revised conclusion now briefly emphasizes that female sex was independently associated with increased odds of rifampicin resistance (Lines 39-42).

Methods

Reviewer Comment 4: Study Design Terminology

Comment:

Please justify whether the term “retrospective cross-sectional study” is methodologically appropriate. Suggested revision: “A cross-sectional study using historical data collected from 2019–2024.”

Response:

We thank the reviewer for this valuable methodological clarification. We agree that the original terminology may create ambiguity regarding the study design. Accordingly, we revised the study design description throughout the manuscript to:

“A cross-sectional study using historical data collected from 2019–2024” (Line 87).

This revised wording more accurately reflects the nature of the analysis while avoiding confusion with retrospective cohort or longitudinal study designs.

Reviewer Comment 5: Need for Trend or Time-Series Analysis

Comment:

If the study is intended to assess temporal trends, more appropriate inferential trend or time-series analyses may be warranted.

Response:

We appreciate this insightful recommendation. Our primary objective was a descriptive assessment of yearly trends in MTB detection and rifampicin resistance using routinely collected diagnostic data rather than formal time-series forecasting or longitudinal modeling. To address the reviewer’s concern:

• We clarified in the Methods section that the trend analyses were descriptive in nature.

• We avoided using terminology suggestive of formal longitudinal or time-series analytical methods.

• Topic has been revised

• We revised figure captions and manuscript wording accordingly.

We also acknowledged in the Discussion (Lines 256-259) and Limitations sections (Lines 284-286) that more advanced time-series analyses could provide additional epidemiological insights in future studies.

Results Section

Reviewer Comment 6: Textual Presentation

Comment:

Suggested revision for line #120:

“Males constituted 56.7% (3,796) of the total tests conducted.”

Response:

Thank you for this suggestion. The sentence has been revised as recommended for improved clarity and readability (Line 118).

Reviewer Comment 7: Table 1 Denominators

Comment:

Please clarify the denominators used for each variable.

Response:

We appreciate this important observation regarding data completeness and denominator consistency. Table 1 has been revised to clearly indicate the denominators for each variable separately. Specifically:

• Sex data were available for 6,698 participants.

• Age data were available for 6,671 participants.

The table headings and corresponding percentages have been corrected accordingly to avoid confusion.

Reviewer Comment 8: Geographic Information

Comment:

If geographic data are available, mapping hotspot areas could support targeted interventions.

Response:

Thank you for this excellent suggestion. Unfortunately, detailed geographic variables such as district, village, or residential location were not consistently available in the dataset extracted from the GeneXpert records. We have now explicitly stated this limitation in the revised manuscript and acknowledged that future studies incorporating geospatial analyses could help identify transmission hotspots and inform targeted TB interventions (275-278).

Reviewer Comment 9: Table 3 Formatting

Comment:

Please check alignment and formatting of superscripts/annotations.

Response:

We thank the reviewer for noting this formatting issue. The superscripts and annotations in Table 3 have been corrected and properly aligned in the revised manuscript.

Reviewer Comment 10: Regression Analysis for MTB Positivity

Comment:

It may also be important to conduct regression analysis for factors associated with MTB positivity.

Response:

We appreciate this valuable suggestion. Due to limitations in available explanatory variables within the dataset, we were unable to perform a robust multivariable analysis for MTB positivity. This limitation has now been acknowledged in the revised manuscript.

Figures

Reviewer Comment 11: Figure 1

Comment:

Panel “C” alone may be sufficient. Avoid including years 2018, 2025, and 2026. The term “longitudinal line graph” is inappropriate.

Response:

We thank the reviewer for these valuable suggestions. While Panel C effectively illustrates the annual changes in MTB grade distribution, we elected to retain Panels A and B because they provide important complementary information on the overall burden of MTB positivity and the cumulative distribution of MTB grades within the study population. Presenting both the aggregate distributions (Panels A and B) alongside the yearly patterns (Panel C) offers readers a more comprehensive understanding of the epidemiological characteristics of MTB in the study setting.

In response to the reviewer's concern, we have removed the years 2018, 2025, and 2026 from the X-axis to improve clarity and have revised the figure title and legend to avoid the use of the term "longitudinal line graph." The figure now describes the annual patterns in MTB grade distribution across the study period.

Reviewer Comment 12: Figure 2

Comment:

Include all years on the X-axis and reconsider the title wording.

Response:

We appreciate the reviewer's suggestion. However, we elected not to include all years on the X-axis because doing so would reduce the clarity and readability of the figure, particularly given the limited plotting space and the number of observations presented. We intended to optimize visual interpretation by emphasizing the overall direction and pattern of rifampicin resistance over the study period as recommended by most researchers and reviewers. Nevertheless, to improve clarity, we have revised the figure title and ensured that the corresponding years are clearly described in the figure legend and Results section.

Variables and Data Completeness

Reviewer Comment 13: Additional Variables

Comment:

Please clarify whether additional variables were available.

Response:

Thank you for this important comment. We clarified in the Methods and Limitations sections that several potentially important variables were unavailable in the extracted dataset, including:

• HIV status

• Treatment history

• Type of TB (PTB/EPTB)

• Specimen type

• Urban/rural residence

• District or village of residence

We have now expanded the limitations section to explicitly discuss how the absence of these variables may have affected the depth of analysis and interpretation. (Lines 275-278).

Discussion

Reviewer Comment 14: Summary of Key Findings

Comment:

Please summarize key findings early in the Discussion.

Response:

We appreciate this recommendation. The opening section of the Discussion has been revised (Lines 174-200) to include a concise summary of the key findings, including:

• Overall MTB detection burden

• Increasing rifampicin resistance trend

• Association between female sex and rifampicin resistance

• Association with low bacillary load

This provides a clearer context for the subsequent interpretation of findings.

Reviewer Comment 15: Temporal Trend in MTB Detection

Comment:

Discuss possible explanations for increasing MTB detection up to 2022 followed by decline afterward.

Response:

Thank you for this insightful comment. We expanded the Discussion section (Lines 181-200) to explore possible explanations for the observed trend, including:

• Expanded GeneXpert testing capacity and intensified case-finding activities before 2022

• Increased public health attention to TB following disruptions during the COVID-19 pandemic

• Possible post-pandemic reductions in healthcare utilization and TB screening activities

• Changes in referral patterns and diagnostic coverage

We also acknowledged that the observed decline may reflect health-system and epidemiologic dynamics requiring further investigation.

Reviewer Comment 16: Female Sex and Rifampicin Resistance

Comment:

The observed association between female sex and rifampicin resistance deserves deeper discussion.

Response:

We appreciate this important observation. The Discussion section has been expanded to more thoroughly interpret the association between female sex and rifampicin resistance (Line 224-240). We discussed several potential explanations, including:

• Delayed healthcare-seeking behavior among women

• Gender-related barriers to accessing TB services

• Socioeconomic inequalities affecting treatment adherence

• Potential delays in diagnosis and initiation of effective therapy

We also highlighted the public health implications for gender-sensitive TB control strategies and equitable access to diagnostic and treatment services in Ghana.

References

Reviewer Comment 17: Reference Accuracy and Use of Primary Studies

Comment:

Please ensure all statements are appropriately referenced and use primary published studies where possible.

Response:

Thank you for this important recommendation. We carefully reviewed and updated the reference list to ensure:

• All statements are appropriately supported by relevant citations.

• Greater use of primary published studies for regional comparisons, including studies from Nigeria, Ethiopia, South Africa, and other Sub-Saharan African countries.

• Consistency in citation formatting according to PLOS ONE guidelines.

All references were cross-checked for accuracy and completeness.

Closing Statement

We sincerely thank the reviewer for the detailed and constructive feedback. The comments have significantly strengthened the scientific quality, clarity, and interpretability of the manuscript. We believe the revised manuscript has substantially improved and hope it is now suitable for consideration for publication in PLOS ONE.

Thank you

Solomon Wireko (PHD)

On behalf of all the authors

Attachment Submitted filename: RESPONSE TO REVIEWERS.docx

# Peer review: Decision Letter 1

**DOI:** 10.1371/journal.pone.0346447.r003

**Authors:** Padmapriya Banada

10 Aug 2026

Dear Dr. Wireko,

Thank you for submitting your manuscript to PLOS ONE. After careful consideration, we feel that it has merit but does not fully meet PLOS ONE’s publication criteria as it currently stands. Therefore, we invite you to submit a revised version of the manuscript that addresses the points raised during the review process.

==============================

Dear authors,

Upon my review of the response to the review questions and the reviewers comments, I think the reviewer 1's comments are valid and I recommend that the authors consider those points carefully, especially regarding the diagnostic algorithm used in this study, which seems to be unclear as presented. I hope you find these comments useful to improve your manuscript further.

==============================

Please submit your revised manuscript by Sep 24 2026 11:59PM. If you will need more time than this to complete your revisions, please reply to this message or contact the journal office at plosone@plos.org. When you're ready to submit your revision, log on to https://www.editorialmanager.com/pone/ and select the 'Submissions Needing Revision' folder to locate your manuscript file.

- A letter that responds to each point raised by the academic editor and reviewer(s). You should upload this letter as a separate file labeled 'Response to Reviewers'.
- A marked-up copy of your manuscript that highlights changes made to the original version. You should upload this as a separate file labeled 'Revised Manuscript with Track Changes'.
- An unmarked version of your revised paper without tracked changes. You should upload this as a separate file labeled 'Manuscript'.

If you would like to make changes to your financial disclosure, please include your updated statement in your cover letter. Guidelines for resubmitting your figure files are available below the reviewer comments at the end of this letter.

If applicable, we recommend that you deposit your laboratory protocols in protocols.io to enhance the reproducibility of your results. Protocols.io assigns your protocol its own identifier (DOI) so that it can be cited independently in the future. For instructions see: https://journals.plos.org/plosone/s/submission-guidelines#loc-laboratory-protocols. Additionally, PLOS ONE offers an option for publishing peer-reviewed Lab Protocol articles, which describe protocols hosted on protocols.io. Read more information on sharing protocols at https://plos.org/protocols?utm_medium=editorial-email&utm_source=authorletters&utm_campaign=protocols.

As the corresponding author, your ORCID iD is verified in the submission system and will appear in the published article. PLOS supports the use of ORCID, and we encourage all coauthors to register for an ORCID iD and use it as well. Please encourage your coauthors to verify their ORCID iD within the submission system before final acceptance, as unverified ORCID iDs will not appear in the published article. *Only*  the individual author can complete the verification step; PLOS staff *cannot*  verify ORCID iDs on behalf of authors.

We look forward to receiving your revised manuscript.

Kind regards,

Padmapriya P Banada, PhD

Academic Editor

PLOS One

Journal Requirements:

If the reviewer comments include a recommendation to cite specific previously published works, please review and evaluate these publications to determine whether they are relevant and should be cited. There is no requirement to cite these works unless the editor has indicated otherwise.

Please review your reference list to ensure that it is complete and correct. If you have cited papers that have been retracted, please include the rationale for doing so in the manuscript text, or remove these references and replace them with relevant current references. Any changes to the reference list should be mentioned in the rebuttal letter that accompanies your revised manuscript. If you need to cite a retracted article, indicate the article’s retracted status in the References list and also include a citation and full reference for the retraction notice.

Additional Editor Comments :

Dear authors,

Upon my review of the response to the review questions and the reviewers comments, I think the reviewer 1's comments are valid and I recommend that the authors consider those points carefully, especially regarding the diagnostic algorithm used in this study, which seems to be unclear as presented. I hope you find these comments useful to improve your manuscript further.

[Note: HTML markup is below. Please do not edit.]

Reviewers' comments:

Reviewer's Responses to Questions

**Comments to the Author**

Reviewer #1: (No Response)

Reviewer #2: All comments have been addressed

**********

2. Is the manuscript technically sound, and do the data support the conclusions?

Reviewer #1: Yes

Reviewer #2: Yes

**********

3. Has the statistical analysis been performed appropriately and rigorously?

Reviewer #1: Yes

Reviewer #2: Yes

**********

4. Have the authors made all data underlying the findings in their manuscript fully available?

The PLOS Data policy

Reviewer #1: Yes

Reviewer #2: Yes

**********

5. Is the manuscript presented in an intelligible fashion and written in standard English?

Reviewer #1: Yes

Reviewer #2: Yes

**********

Reviewer #1: This study if of high importance to local and national health authorities expressing a considerable health issue in highlighting TB under-detection and increased drug resistance in the studied geographical area. Though to better express self and finding, following comments need to be considered:

Corrections should be carefully addressed in both article and abstracts.

Introduction:

Authors did not give information about the TB/RRTB incidence or burden in the geographical area under study in the area, so as to compare study findings. Apparently, there is an outbreak of RRTB in this area according to this study (17.7% out of confirmed TB??). this should be judged in light of knowledge about the structure of TB program in studied geographic are including issues of under-detention/underreporting, and TB program TOR of other health facilities.

Design:

Authors should unify the study design in both article text in the abstract: “A cross-sectional study using historical data collected from 2019–2024” (Line 87).

Case definitions: This study is missing the definitions of presumptive TB, and MTB detection, RR detection. Did this study include other than sputum samples or other than pulmonary disease?

It is not clear in this study text how much this hospital is important in expressing TB control/surveillance efforts in Ghana as there is no/insufficient narration about each of:

1- How much residential people represent out of Ghana?

2- Other health facilities in the drainage area role in TB detention/surveillance?

It is not clear in this study text how much hospital bias is contributing to observed findings as there is no/insufficient narration about each of

1. How much the sample represent the population (presumptive TB) in this referral hospital?

2. How much the GX covered presumptive cases? Algorithm for MTB/RRTB diagnosis in this hospital?

3. Is there a contribution over years to the GX kits stocking/machine functioning to the observed fluctuation? or it is merely a matter of referral system activation?

To estimate predictors, the investigators used adjusted multivariate logistic regression but not adjusted OR tests

aOR? Adjusted for what?? For females it is adjusted for age and for bacterial load it is adjusted for age and sex.

Repeat tests within 30 days for 94 the same patient were excluded to minimize duplication. Is this cross-checking enough? GX Repeats and follow-up test should be excluded for all enrolled persons.

The test used is chi-square for independence (there are other types of chi-square tests)

Results:

Tables 2&3 did not express themselves well, should readers assume that presented bare number are absolute and those between brackets are percentages?

Conclusion

The main conclusion is in the fluctuation of referred presumptive cases to this hospital

The fluctuation is in the referral system activation (perhaps to the variation in under-detection or underreporting) rather than in the positivity

Significant temporal variation?? Direction should be expressed?

Other conclusions should confine to hospital cases not to the geographical area patients. (issues in results generalizability)

High RR rate?? 17.7%. This need to be thoroughly discussed before conclude it in this study. This finding call for a huge and urgent action to stop further progression of RR burden.

Authors needn’t to add recommendations to the abstract, but if so they can brief it into “serious investigation should go to verify both the causes behind MTB detection fluctuation and the existence and causes of high RRTB burden in studied area”

Mentioned Limitations are not major limitations for this study particularly for the use of GX for TB/RRTB detection as the study used a highly sensitive technique and the study aimed at studying the time trend of detection rather that the predictors of detection.

Reviewer #2: The conclusions are well supported by the data presented. The increasing trend in resistance observed over recent years, together with the independent associations of female sex and low bacterial load with rifampicin resistance, are consistent with the study findings. Of particular interest is the association between low bacterial load and rifampicin resistance, which represents a noteworthy finding and warrants further consideration in the discussion.

**********

what does this mean?). If published, this will include your full peer review and any attached files.

If you choose “no”, your identity will remain anonymous but your review may still be made public.

**Do you want your identity to be public for this peer review?**  For information about this choice, including consent withdrawal, please see our Privacy Policy

Reviewer #1: **Yes:**  LAYTH AL-SALIHI

Reviewer #2: No

**********

[NOTE: If reviewer comments were submitted as an attachment file, they will be attached to this email and accessible via the submission site. Please log into your account, locate the manuscript record, and check for the action link "View Attachments". If this link does not appear, there are no attachment files.]

To ensure your figures meet our technical requirements, please review our figure guidelines: https://journals.plos.org/plosone/s/figures

You may also use PLOS’s free figure tool, NAAS, to help you prepare publication quality figures: https://journals.plos.org/plosone/s/figures#loc-tools-for-figure-preparation.

NAAS will assess whether your figures meet our technical requirements by comparing each figure against our figure specifications.

# Author comment: Author response to Decision Letter 2

**DOI:** 10.1371/journal.pone.0346447.r004

19 Aug 2026

Response to Reviewer 1

We sincerely thank Reviewer 1 for the careful and constructive second-round review of our manuscript entitled “Annual Trends in Mycobacterium tuberculosis Detection and Rifampicin Resistance at a Regional Referral Hospital in Northern Ghana, 2019–2024.” We appreciate the reviewer’s recognition of the study's public health importance and the valuable suggestions aimed at improving the epidemiological interpretation, methodological clarity, and generalizability of our findings.

We have carefully considered all comments and have revised the manuscript accordingly. In particular, we have clarified that the study describes hospital-based GeneXpert testing trends rather than population-level TB incidence, strengthened the description of the diagnostic and referral context, refined the interpretation of rifampicin resistance, clarified the multivariable logistic regression analysis, improved the presentation of Tables 2 and 3, and revised the conclusion and limitations to ensure that our claims remain proportionate to the study design.

For clarity, each comment is reproduced below, followed by our response and the corresponding changes made to the manuscript.

Comment 1: Corrections should be carefully addressed in both article and abstracts.

Response:

Thank you for this important observation. We carefully reviewed both the Abstract and the main manuscript to ensure consistency in terminology, study design, interpretation of the findings, and the scope of the conclusions.

In particular, we have revised the Abstract to avoid implying that the observed proportions represent population-level TB incidence or prevalence in the Upper East Region. The revised Abstract now explicitly identifies the study as a retrospective analysis of GeneXpert MTB/RIF records from a regional referral hospital and presents the 17.7% rifampicin-resistance estimate specifically as the proportion observed among MTB-confirmed samples tested at the hospital (Lines 45-47).

We have also revised the concluding statement of the Abstract to emphasize temporal variation in hospital-based detection and the need for further investigation rather than making a direct inference about the population-level burden of rifampicin-resistant TB in Northern Ghana (Lines 48- 50).

Comment 2: Introduction: Authors did not give information about TB/RRTB incidence or burden in the geographical area under study. The apparent 17.7% RR-TB finding should be judged in light of the TB programme structure, under-detection/underreporting, and the terms of reference of other health facilities.

Response:

We agree. The previous version provided national TB estimates but insufficient regional context. We have therefore expanded the Introduction and Discussion to distinguish between:

1. national estimates of TB and MDR/RR-TB;

2. regional/hospital-based surveillance data; and

3. the estimates generated by the present study.

The 17.7% estimate is calculated as 192/1,083 among MTB-positive GeneXpert samples tested at the Upper East Regional Hospital and should therefore not be interpreted as the incidence or prevalence of RR-TB in the Upper East Region or Northern Ghana. The denominator consists of MTB-confirmed samples reaching this particular diagnostic facility and is consequently influenced by referral patterns, case-finding practices, clinical selection of patients for GeneXpert testing, and access to molecular diagnostics.

We have revised the manuscript to make this distinction explicit and to avoid describing the finding as evidence of an outbreak.

The 2021 Ghana Population and Housing Census reports a population of approximately 1.30 million for the Upper East Region, representing approximately 4% of Ghana's population. We have used this information only to provide population context and have explicitly stated that our hospital-based dataset cannot be converted into a regional population prevalence estimate.

We have also expanded the discussion of the National Tuberculosis Control Programme and the role of referral facilities. Because the present dataset does not contain complete information on the number and characteristics of presumptive TB patients managed at every facility in the region, we have avoided claiming that the observed pattern represents the entire regional TB programme.

Change made: The Introduction and Discussion now explicitly state that the study provides hospital-based surveillance evidence and should not be interpreted as a direct estimate of regional TB incidence or RR-TB prevalence (Lines 98-105 and Lines 112-114)

Comment 3: The study design should be unified in the article and abstract: “A cross-sectional study using historical data collected from 2019–2024.”

Response:

We agree. The study design has now been made consistent throughout the manuscript and Abstract.

The Methods section currently states:

“A cross-sectional study using historical data collected from 2019–2024…”

We have retained this wording and have ensured that the Abstract uses the same terminology. We have also avoided using “longitudinal study” in places where it could imply prospective follow-up of individuals.

Change made: Study design terminology has been standardized throughout the Abstract, Methods, Results, Discussion, and Conclusion (Lines 24-25 and Line 117).

Comment 4: The case definitions are missing. Define presumptive TB, MTB detection, and rifampicin resistance. Did the study include samples other than sputum or patients with extrapulmonary disease?

Response:

We thank the reviewer for highlighting the need for explicit operational definitions. We have now added definitions of presumptive TB, MTB detection, and rifampicin resistance to the Methods section.

The study included sputum specimens only. No other clinical specimen types were included. Consequently, the study population comprised patients evaluated for presumptive pulmonary TB, and extrapulmonary TB was not assessed.

Presumptive TB is now defined as a patient clinically evaluated for possible pulmonary TB for whom a sputum specimen was submitted for GeneXpert MTB/RIF testing. MTB detection is defined as a GeneXpert result reporting detection of Mycobacterium tuberculosis complex, while rifampicin resistance is defined as GeneXpert detection of mutations associated with rifampicin resistance within the rpoB target region (Lines 131-139).

We have also clarified that GeneXpert-detected rifampicin resistance represents a molecular marker of rifampicin resistance and does not, by itself, establish resistance to isoniazid or constitute a complete phenotypic drug-susceptibility profile.

Change made:

The Methods section has been revised to explicitly state that only sputum specimens were included and that the study therefore evaluated pulmonary TB only (Lines 140-141).

Comment 5: The importance of this hospital for TB surveillance in Ghana is unclear. How much of the regional population does it represent?

Response:

We appreciate this point and have expanded the description of the study setting.

The Upper East Regional Hospital is a regional referral facility in Bolgatanga and functions as a diagnostic and treatment centre for TB under the Ghana National Tuberculosis Control Programme. The study therefore provides an important source of routine molecular surveillance data for patients referred to or tested at the facility.

However, we agree that the hospital's GeneXpert records cannot be assumed to represent the entire regional population. The Upper East Region had a population of approximately 1.30 million in the 2021 Population and Housing Census, representing approximately 4% of Ghana's population.

We have consequently revised the manuscript to avoid implying that the hospital dataset represents all residents of the Upper East Region.

The revised manuscript now emphasizes that the study provides evidence from a regional referral diagnostic facility, rather than a population-based surveillance system (Lines 121-126).

Comment 6: Other health facilities in the drainage/catchment area and their role in TB detection and surveillance are insufficiently described.

Response:

We agree that the referral network is important when interpreting the observed temporal variation.

Patients suspected of TB may enter the diagnostic pathway through different levels of the health system, and the number of patients ultimately tested at the regional referral hospital may vary according to referral practices, availability of GeneXpert testing at peripheral facilities, clinical suspicion, and programme-directed case-finding.

The present dataset, however, does not contain sufficiently detailed information on all presumptive TB patients assessed at other health facilities in the region or on the number subsequently referred to the Upper East Regional Hospital. We therefore cannot quantify the proportion of all regional presumptive TB cases captured by this hospital.

We have now explicitly acknowledged this issue and have reframed the interpretation of annual fluctuations as variation in hospital-based diagnostic activity and referral patterns, rather than assuming equivalent changes in the underlying regional incidence of TB (Lines 266- 274).

Comment 7: Hospital bias is unclear. How representative is the sample of presumptive TB patients attending the hospital?

Response:

We agree that this is an important limitation.

The 6,897 records represent GeneXpert MTB/RIF tests performed at the Upper East Regional Hospital during the study period; they do not represent all individuals who attended the hospital with respiratory symptoms or all persons with presumptive TB in the hospital catchment area.

The denominator therefore represents patients who were selected for and underwent GeneXpert testing, rather than the complete population of presumptive TB patients.

We have revised the Methods and Discussion to make this distinction explicit. We have also clarified that selection for GeneXpert testing may have been influenced by clinical suspicion, referral status, previous TB treatment, risk of drug resistance, or programme-specific testing criteria (266-269).

Accordingly, the observed MTB positivity and RR proportions should be interpreted as testing-population estimates rather than unbiased population estimates.

Comment 8: How much did GeneXpert cover presumptive TB cases? What was the diagnostic algorithm?

Response:

We thank the reviewer for raising this important issue. We have clarified the diagnostic pathway in the revised Methods (Line 144-147 and Lines 151-155).

At the Upper East Regional Hospital, GeneXpert MTB/RIF was used as the initial diagnostic test for presumptive pulmonary TB, and testing was not prioritized for particular patient groups. All presumptive TB patients who entered the hospital's diagnostic pathway and provided the required sputum specimen were therefore eligible for initial GeneXpert testing.

All specimens included in the present study were sputum specimens. Patients with MTB detected by GeneXpert, including those with rifampicin resistance detected, were subsequently referred for culture and drug-susceptibility testing (DST).

We have also clarified an important distinction regarding GeneXpert coverage. Although GeneXpert was the initial diagnostic test for presumptive TB at UERH, the retrospective dataset does not contain a complete denominator of every person clinically assessed as presumptive TB in the hospital or the wider regional health system. We therefore cannot calculate the proportion of all presumptive TB patients in the Upper East Region who were represented in the dataset.

Because testing was not preferentially restricted to particular high-risk groups, we believe the possibility of deliberate selection of patients based on known risk of drug resistance is reduced. Nevertheless, referral and healthcare-seeking patterns may still influence the composition of patients reaching a regional referral hospital. We have therefore retained this consideration when interpreting the 17.7% rifampicin-resistance proportion.

Change made:

The Methods section now explicitly describes GeneXpert MTB/RIF as the initial diagnostic test for presumptive pulmonary TB, states that testing was not prioritized for specific patient groups, confirms that only sputum specimens were included, and describes referral of MTB-positive and rifampicin-resistant cases for culture and DST.

(Lines 142-145 and Line 149-153).

Comment 9: Could fluctuations be due to GeneXpert cartridge supply, machine functionality, or changes in the referral system?

Response:

We agree that this is an important alternative explanation for the observed temporal variation.

The current dataset does not contain complete machine-maintenance logs, cartridge stock records, downtime records, or annual documentation of referral activation. Therefore, we cannot determine whether the observed changes in the number of tests and MTB detections were caused by changes in cartridge availability, instrument functionality, referral activity, case-finding intensity, or healthcare-seeking behaviour.

We have therefore revised the Discussion to avoid attributing the temporal fluctuations to a specific cause.

The revised interpretation is that the observed fluctuations may reflect a combination of changes in diagnostic access, referral activity, case-finding practices, healthcare utilization, and health-system disruptions, including the COVID-19 period (Lines 266-274).

We now explicitly state that these mechanisms cannot be distinguished using the available retrospective dataset and warrant further investigation.

Comment 10: “Adjusted multivariate logistic regression but not adjusted OR tests. aOR? Adjusted for what? For females it is adjusted for age and for bacterial load it is adjusted for age and sex.”

Response:

We thank the reviewer for identifying this ambiguity.

We have clarified the statistical analysis and interpretation of the adjusted odds ratios.

The multivariable logistic regression model included age group, sex, and MTB bacterial-load category. Accordingly:

• the aOR for female sex represents the odds of rifampicin resistance adjusted for age group and MTB bacterial-load category; and

• the aOR for low MTB grade represents the odds of rifampicin resistance adjusted for age group and sex.

The previous wording suggesting that the female association was adjusted for sex was incorrect and has been removed.

The revised Results section now states:

“In the multivariable logistic regression model, female sex was associated with higher odds of rifampicin resistance after adjustment for age group and MTB bacterial-load category (aOR = 1.44; 95% CI: 1.02–2.02; p = 0.037). Low MTB bacterial load was independently associated with rifampicin resistance after adjustment for age group and sex (aOR = 9.62; 95% CI: 5.77–16.04; p < 0.001)” (Lines 233-239).

This is also reflected in the revised Table 3 footnote.

Comment 11: Repeat tests within 30 days for 94 patients were excluded. Is this cross-checking sufficient? Repeat and follow-up GeneXpert tests should be excluded for all enrolled persons.

Response:

We thank the reviewer for this important observation. In the original data-cleaning procedure, repeat GeneXpert tests from the same patient occurring within 30 days were identified and excluded to minimize duplication. A total of 94 such repeat tests were excluded.

We acknowledge that a 30-day criterion may not identify every possible follow-up or repeat test occurring after 30 days. However, because the original retrospective dataset and analysis were constructed using this prespecified data-cleaning criterion, and because complete longitudinal linkage of all patient records beyond this interval was not available, we have retained the original analytical dataset rather than retrospectively modifying the inclusion criteria without the ability to reproduce the complete analysis.

We have clarified this point in the revised Methods and Limitations sections. We also acknowledge that some follow-up tests occurring outside the 30-day interval may not have been identifiable as duplicates, and this should be considered when interpreting the findings.

Change made:

The Methods section now states:

“To minimize duplicate observations, repeat

Attachment Submitted filename: RESPONDS TO REVIEWERS.docx

# Editor report: Decision Letter 2

**DOI:** 10.1371/journal.pone.0346447.r005

**Authors:** Padmapriya Banada

26 Aug 2026

<p>Annual Trends in Mycobacterium tuberculosis Detection and Rifampicin Resistance at a Regional Referral Hospital in Northern Ghana, 2019–2024

PONE-D-26-13133R2

Dear Dr. Wireko,

We’re pleased to inform you that your manuscript has been judged scientifically suitable for publication and will be formally accepted for publication once it meets all outstanding technical requirements.

Within one week, you’ll receive an e-mail detailing the required amendments. When these have been addressed, you’ll receive a formal acceptance letter and your manuscript will be scheduled for publication.

An invoice will be generated when your article is formally accepted. Please note, if your institution has a publishing partnership with PLOS and your article meets the relevant criteria, all or part of your publication costs will be covered. Please make sure your user information is up-to-date by logging into Editorial Manager at Editorial Manager® and clicking the ‘Update My Information' link at the top of the page. For questions related to billing, please contact billing support.

If your institution or institutions have a press office, please notify them about your upcoming paper to help maximize its impact. If they’ll be preparing press materials, please inform our press team as soon as possible -- no later than 48 hours after receiving the formal acceptance. Your manuscript will remain under strict press embargo until 2 pm Eastern Time on the date of publication. For more information, please contact onepress@plos.org.

Kind regards,

Padmapriya P Banada, PhD

Academic Editor

PLOS One

Additional Editor Comments (optional):

Dear Authors,

Thank you for considering the reviewer's comments and appreciate your responses improving the manuscript significantly. I am happy to now recommend your manuscript for publication in PlosOne.

Best regards

Reviewers' comments:

# Editor report: Acceptance letter

**DOI:** 10.1371/journal.pone.0346447.r006

**Authors:** Padmapriya Banada

PONE-D-26-13133R2

PLOS One

Dear Dr. Wireko,

I'm pleased to inform you that your manuscript has been deemed suitable for publication in PLOS One. Congratulations! Your manuscript is now being handed over to our production team.

At this stage, our production department will prepare your paper for publication. This includes ensuring the following:

* All references, tables, and figures are properly cited

* All relevant supporting information is included in the manuscript submission,

* There are no issues that prevent the paper from being properly typeset

You will receive further instructions from the production team, including instructions on how to review your proof when it is ready. Please keep in mind that we are working through a large volume of accepted articles, so please give us a few days to review your paper and let you know the next and final steps.

Lastly, if your institution or institutions have a press office, please let them know about your upcoming paper now to help maximize its impact. If they'll be preparing press materials, please inform our press team within the next 48 hours. Your manuscript will remain under strict press embargo until 2 pm Eastern Time on the date of publication. For more information, please contact onepress@plos.org.

You will receive an invoice from PLOS for your publication fee after your manuscript has reached the completed accept phase. If you receive an email requesting payment before acceptance or for any other service, this may be a phishing scheme. Learn how to identify phishing emails and protect your accounts at https://explore.plos.org/phishing.

If we can help with anything else, please email us at customercare@plos.org.

Thank you for submitting your work to PLOS One and supporting open access.

Kind regards,

PLOS One Editorial Office Staff

on behalf of

Dr. Padmapriya P Banada

Academic Editor

PLOS One
